# Ethnobotany, Phytochemistry, and Biological Activity of Extracts and Non-Volatile Compounds from *Lantana camara* L. and Semisynthetic Derivatives—An Updated Review

**DOI:** 10.3390/molecules30040851

**Published:** 2025-02-12

**Authors:** Jorge Ramírez, Chabaco Armijos, Nelson Espinosa-Ortega, Leydy Nathaly Castillo, Giovanni Vidari

**Affiliations:** 1 Departamento de Química, Facultad de Ciencias Exactas y Naturales, Universidad Técnica Particular de Loja, Loja 1101608, Ecuador; cparmijos@utpl.edu.ec (C.A.); lncastillo@utpl.edu.ec (L.N.C.); 2 UTPL-Alumni, Barrio San Cayetano Alto, Calle Marcelino Champagnat, Loja 1101608, Ecuador; njespinosa1@utpl.edu.ec; 3 Department of Medical Analysis, Faculty of Applied Science, Tishk International University, Erbil 44001, Iraq; vidari@unipv.it

**Keywords:** Verbenaceae, *Lantana camara*, ethnobotany, phytochemistry, biological activity

## Abstract

*Lantana camara* L., commonly known as pigeon berry, is a herbaceous plant of growing scientific interest due to the high medicinal value. In fact, despite being categorized as an invasive species, it has been used for a long time to treat different diseases thanks to the many biological activities. Triterpenes, flavonoids, phenylpropanoids, and iridoid glycosides are the bioactive compounds naturally occurring in *L. camara* that have demonstrated anticancer, antifilarial, nematocidal, antibacterial, insecticidal, antileishmanial, antifungal, anti-inflammatory, and antioxidant properties. The aim of this review is to update the information concerning the chemistry and biological activity of *L. camara* extracts and their constituents, including semisynthetic derivatives, revising the literature until June 2024. We believe that the data reported in this review clearly demonstrate the importance of the plant as a promising source of medicines and will therefore stimulate further investigations.

## 1. Introduction

According to the latest Angiosperm Phylogeny Group classification (APG IV), the genus *Lantana* L. is one of the 32 genera belonging to the family Verbenaceae J. St. Hill., in the order Lamiales [1]. This genus comprises 133 species with accepted names according to the December 2023 WFO classification [2] (latest access: 31 May 2024). However, this number may change in the future due to the description of new species or the segregation into other genera [3]. Moreover, the taxonomic classification of the genus is difficult, since species are not stable and widespread hybridization occurs, while morphological characters vary with age.

A recent phylogenetic study of the tribe Lantaneae Endl. stated that the genus *Lantana* L. is not monophyletic and placed the species *Lantana camara* L. into the section Lantana sect. Lantana together with *L*. *horrida* Kunth, *L. depressa* Small, *L*. *leonardiorum* Moldenke, *L. sabrida* Sol., and *L. strigocamara* R.W. Sanders [4].

**Figure 1:**
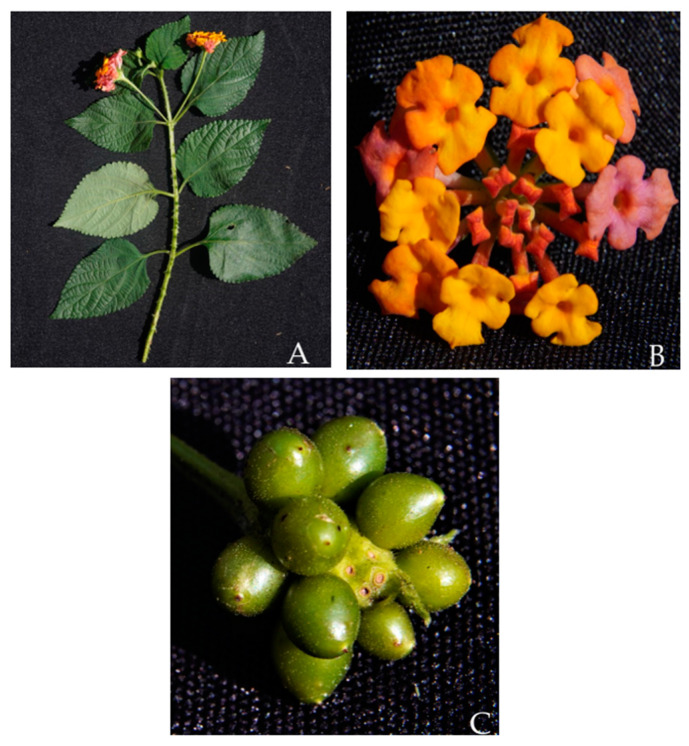
*Lantana camara* L.: (**A**) entire plant; (**B**) flowers; (**C**) fruits (photos by the authors).

**Figure 2:**
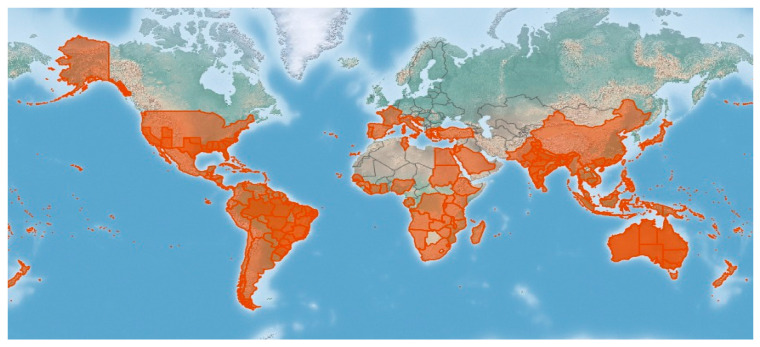
Worldwide distribution of *Lantana camara* L. [11].

*L. camara* L., which is the most widespread species of the genus, is an evergreen aromatic spiny hairy shrub, usually 0.5–3 m high (Figure 1), bearing flowers of different colors, from red to pink, white, yellow, orange, and violet. The stems and branches are sometimes armed with prickles or spines; the leaves are opposite, simple with large petioles, and oval blades, which are rugged and hairy and have a bluntly toothed margin. The plant is known by different popular names, such as pigeon berry [5], wild red sage [6], cuasquito, angel lip, flowered sage, black sage, shrub verbena, white sage, and wild sage [7]. It is native to tropical and subtropical Central and South America, from where it was introduced to other countries, and it has spread all over the world [8] (Figure 2). *L. camara* is considered an invasive obnoxious weed of pastures, orchards, and forest areas, as well as a cultivated ornamental or garden hedge plant [9,10,11]. The poisonous properties of the plant have been known for a long time, especially to livestock; on the other hand, toxicity to humans from fruit ingestion has also been reported. Due to the plant cosmopolitan distribution and the innate ability to produce hybrids, some varieties and subspecies are known [12], and have been proposed in a taxonomic revision of *Lantana* L. sect. *Lantana* [13]. However, because of the intrinsic taxonomic complexity [14], in this review, all of the subspecies and varieties have not been treated separately, but have been incorporated into a single species, *L. camara* L. sensu lato.

*L. camara* is one of the most important herbal medicines in the world. For example, it is well known in the Ayurvedic medicinal system with the Sanskrit names of Chaturangi and Vanacchedi. Different parts of the plant are used as traditional remedies for the treatment of various human ailments, such as itches, cuts, ulcers, swellings, bilious fever, catarrh, asthma and bronchitis, eczema, chicken pox, tetanus, malaria, tumors, stomachache, toothache, headache, scabies, leprosy, rheumatism, and as an antiseptic agent to treat wounds [4,5,15,16]. The essential oil has shown antibacterial, antifungal, cytotoxic, and mosquito-repellent effects. *L. camara* has been found to display a variety of biological properties, including antiarthritic, anti-aspergillus, antibacterial, anticancer, cardioactive, anti-fertility, antifilarial, hepatoprotective, anti-hyperglycemic, anti-hyperlipidemic, anti-inflammatory, insecticidal, antimicrobial, antimutagenic, anxiolytic, nematocidal, antioxidant, anti-proliferative, anti-protozoal, antipyretic, antithrombin, antitumor, antiulcerogenic, antiurolithiasis, antiviral, and wound-healing properties. Moreover, the plant extracts have been reported to inhibit the enzymes acetylcholinesterase, alpha amylase, carboxylesterase, cyclooxygenase-2, inducible nitric oxide synthase (iNOS), glutathione-S-transferase (GST), 5-lipoxygenase (5-LOX), protein kinase C, and xanthine oxidase [17,18]. Phytochemical studies conducted by different research groups have led to the isolation of essential oils, various steroids, terpenoids, saponins, iridoids, flavonoids, phenylethanoids, naphthoquinones, coumarins, polyphenols and other phenolics, and alkaloids [17,18,19,20,21,22,23]. Interestingly, the genus *Lantana* is free of diterpenoids [17].

The information concerning the phytochemistry and the biological activities of *L. camara* L. published until March 2000 has been condensed in previous reviews [17,18,21,24]. Moreover, recent studies on ecological aspects, chemical constituents, semisynthetic derivatives [25,26,27], and the biological and pharmacological activities of *L. camara* have been reviewed [17,18,20,21,22,23,28,29,30,31,32,33,34,35]. However, these publications, although dealing with different aspects of the plant, are largely incomplete. Therefore, the purpose of this review is to update and complete the information about *L. camara* L. to serve as a starting point for further investigations of the plant.

## 2. Research Strategies and Literature Sources

**Figure 3:**
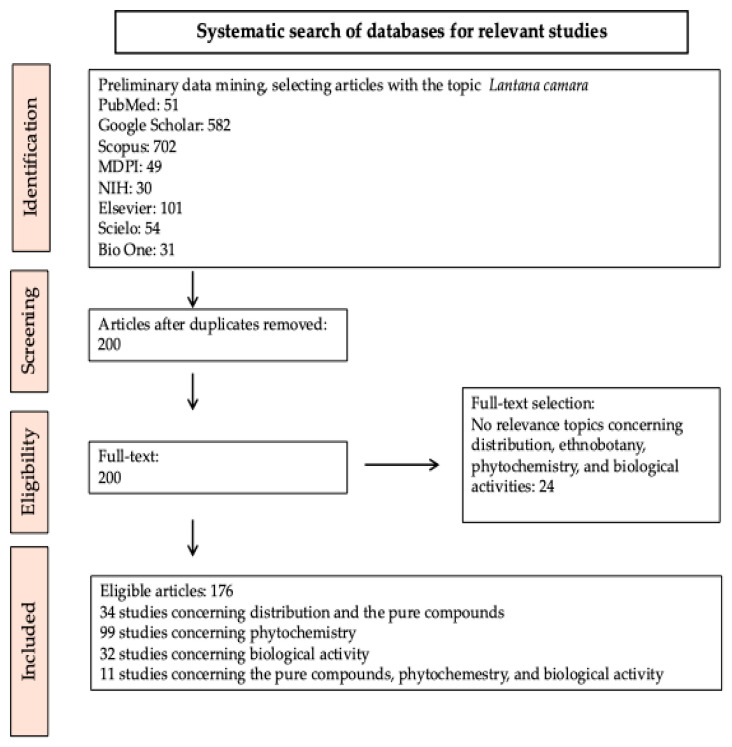
Flowchart for the search process and selection of the studies considered for the review.

To prepare this review, the literature from 14 March 2000 until 10 June 2024 has been retrieved from the following databases: Pub-Med (https://pubmed.ncbi.nlm.nih.gov/, accessed on 10 June 2024), Google Scholar (https://scholar.google.com/, accessed on 9 March 2024), Scopus (https://www.scopus.com/, accessed on 9 March 2024), MDPI (https://www.mdpi.com/, accessed on 9 March 2024), NIH (https://www.nih.gov/, accessed on 9 March 2024), Elsevier (https://www.elsevier.com/, accessed on 9 March 2024), Scielo (https://scielo.org/es/, accessed on 9 March 2024), and Bio One (https://bioone.org/, accessed on 9 March 2024). The most relevant papers dedicated to the phytochemistry and the in vitro and in vivo biological effects of *L. camara* extracts, isolated chemical compounds, and semisynthetic derivatives were initially considered. Subsequently, among the more than 1600 articles published on *L. camara*, the manager software Mendeley Desktop software version 1.19.8 was used led us to select and review the research papers mainly dedicated to the above-mentioned topics. Moreover, all duplicated articles and gray sources were removed. After this first selection, a total of approximately 200 articles directly related to the topics of the present review were further reduced to 176 based on the relevance of the information provided by each of them. The systematic search of databases for relevant articles published on *L. camara* to compile this review is shown in Figure 3.

## 3. Results

### 3.1. Ethnobotany

In Table 1 we have reported the distribution of *L. camara* L. in different regions and countries of the world, together with the local vernacular names, the part of the plant used and the preparation methods of traditional remedies.

**Table 1:** Distribution and traditional uses of *Lantana camara* L. in various continents and countries of the world.

Region	Country	Vernacular Name	Part of the Plant	Preparation Method	Traditional Uses	Ref.
Africa	Congo (Bouenza Department)	Lantana (Kunyi)	Leaves	Decoction	Antidiarrheal	[36]
	Democratic Republic of Congo (Bukavu and Uvira)	Mwamuganga (Mashi), Mavi ya kuku (Swahili), Makereshe (Nande)	Leaves	Decoction	Antimalarial	[37]
	Democratic Republic of Congo (Kisantu and Mbanza-Ngungu)	Nsudi nsudi (Kikongo)	Leaves and fruits	Decoction; administered by rectal route	To treat cough by the Ntându and Ndibu ethnic groups; to treat hemorrhoids	[38]
	Ethiopia (Libo-Kemkem District)	NR	Leaves	Infusion	Antidiarrheal	[39]
	Ethiopia (Mana Angetu District)	NR	Leaves	Decoction	To treat skin infections, gonorrhea, and “evil eye”	[40]
	Ethiopia (Wonago Woreda)	Yewof kollo (Amharic)	Stems	Infusion	Antidiarrheal	[41]
	Ethiopia (Sheko District)	Michi-charo (Sheko)	Leaves	Topical	To treat “Michi”, a type of febrile illness	[42]
	La Réunion	Galabert, Corbeille d’or (French)	Leaves	Decoction, infusion	Antimalarial	[43]
	Guinea (Low, middle, upper, and forest ecological zones)	Tagani (NR)	Leaves	Decoction	Soussous, Malinké, Guerzé, Konon, and Manon ethnic groups use the plant to treat infectious diseases	[44]
	Kenya (Central Province)	Rûîthiki, Mûkenia (Kikuyu)	Leaves	Crushed; directly applied to the ear to treat otitis	Kikuyu ethnic group uses the plant to treat common cold by inhaling crushed leaves	[45,46]
	Kenya (Embu and Mbeere Districts)	Mûkenia (Kikuyu)	Leaves	Decoction	Antimalarial	[47]
	Kenya (Formerly: Bondo District, now Siaya County)	Nyabend winy (Luo)	Leaves and roots	Decoction	To treat cough	[48]
	Kenya (Msambweni District)	Mjsasa (Digo)	Leaves	Decoction	Digos, Durumas, and Kambas ethnic groups use the plant as an antimalarial	[49]
	Kenya (Rusinga Island and Rambira)	NR	Leaves and seeds	Burnt for fumigation	Used as a mosquito repellent	[50]
	Madagascar (Antsiranana)	Kalabera (NR)	Aerial parts	Decoction	To treat cough, hypertension, and fever	[51]
	Nigeria (Ibadan)	Wild sage (English)	Leaves	Burnt for fumigation	Used as an insect repellent	[52]
	Uganda (Budiope County)	Kapanga (Lusoga)	Leaves	Burnt for fumigation; decoction	Used as a mosquito repellent; antimalarial	[53]
	Uganda (Budondo Subcounty)	Kapanga (Lusoga), Tickberry (English)	Leaves	Burnt for fumigation	Used as a housefly and insect repellent	[54]
	Uganda (Otwal and Ngai Subcounties)	NR	Leaves and roots	Maceration	Used to treat ringworms, cataracts, snake bites, and epilepsy	[55]
Asia	China (Xishuangbanna)	Luo-ya-min (Chinese)	Leaves	Burnt for fumigation	Used as a mosquito repellent	[56]
	India (Assam)	Bhoot-phool (Hindi)	Bark	Burnt for fumigation; decoction	Used as an insect repellent; antimalarial	[57]
	India (Dharmapuri District)	NR	Leaves	Decoction, infusion	Antimalarial	[58]
	India (Jharkhand State)	Puttu (NR)	Leaves	Decoction, pounded	To treat several skin and respiratory diseases	[59]
	India (NR)	NR	Leaves	Decoction	Antiseptic, antimalarial, and antirheumatic	[60]
	Philippines (Paroc)	Gainis (NR)	Leaves and stems	Burnt for fumigation	Used as an insect repellent	[61]
	Vietnam (Hướng Hóa District)	Thục Klay (NR)	Roots	Decoction	Van Kieu ethnic group uses the plant alone or associated with roots of *Mangifera indica* and barks of *Erythrina variegata* to treat abdominal pain and diarrhea	[62]
	Yemen (Hajjah District)	NR	Flowers, leaves, and seeds	Burnt for fumigation	Used as an insect repellent	[63]
North and Central America	Mexico (Querétaro)	Alfombrilla, Gobernadora, Ororuz (Spanish)	Leaves and stems	Decoction	To treat scorpion and insect stings; antidiarrheal and antiparasitic	[64]
	Mexico (Chiapas)	Jøtskuy (Zoque), Cinco negritos (Spanish)	Stems	Decoction	Antidiarrheal, antiparasitic, and antirheumatic	[65]
	Mexico (Puebla)	Cinco negritos (Spanish)	Aerial parts	Decoction	Antidiarrheal	[66]
South America	Brazil (Minas Gerais)	Cambará (Tupi)	Leaves	Decoction, infusion	To treat respiratory diseases; antipyretic and antirheumatic	[67]
	Suriname (Pikin Slee)	NR	Leaves	Decoction	The Saramaccan Marron ethnic group uses the plant for the anti-inflammatory, antiparasitic, and depurative properties	[68]
	Colombia (Antioquia Department)	Venturosa (Spanish)	Stems	Decoction; steam bath	To treat snake bites	[69]
The Caribbean	France (Guadeloupe)	Mille-fleurs (French)	Flowers	Decoction, infusion	To treat flu syndrome	[70]

NR: not reported.

### 3.2. Phytochemistry

A total of 168 compounds have been described with different names in the considered period. They include both specialized metabolites isolated from non-volatile fractions of *L. camara* as well as semisynthetic derivatives. The distribution pattern of these compounds includes steroids and triterpenoids (75.6%), flavonoids (14.3%), fatty acids and other miscellaneous compounds (8.9%), and iridoid glucosides (1.2%).

#### Triterpenoids and Steroids

Steroids and pentacyclic triterpenoids are the predominant constituents isolated in the indicated period from the non-volatile fractions of *L. camara* or obtained by semisynthesis. Steroids (Table 2) are only a few and include common phytosterols such as stigmasterol (**1**), β-sitosterol (**3**), and campesterol (**5**), in addition to the rare spirostane saponin yamogenin II (**6**), which has a unique aglycone moiety. One hundred and twenty-one triterpenoids (Table 3) have been described in the years considered in this review. Their molecular structures belong to only five families, i.e., the protostane, euphane, lupane, oleanane, and ursane ones. The last two skeletons are by far the most common. Alisol A (**7**) is the only protostane isolated from *L. camara*, while euphanes are represented by eight triterpenoids (**8–15**). The structures of most of them are characterized by a D^7^ double bond, an oxidized α-substituent at C-4, and a γ-lactone E ring that is *trans*-fused to the cyclopentane D ring and bears an unsaturated homoprenyl chain. The small lupane family (**16–18**) includes the rare lantabetulic acid (**17**), which is characterized by an ether β-bridge connecting C-3 with C-25, and the highly bioactive betulinic acid (**18**). A total of 78 oleanane triterpenoids (**19–96**), including 34 synthetic ones, and 31 ursane derivatives (**97–127**), including 6 synthetic ones, have been described in the considered period. The two skeletons differ from the position of the methyl groups C-29 and C-30, which are positioned on the quaternary carbon C-20 in the oleanane compounds, while they are *trans*-oriented on the tertiary carbons C-19 and C-20 in the ursane derivatives.

The great variety of oleanane triterpenoids from *L. camara* derive from a combination of differently placed double bonds and different oxygenated groups that decorate the basic skeleton. A β-COOH, as in compound **30**, or a β-COOMe group, as in **33**, is usually linked to C-17, with *cis*-orientation to βH-18. When a carboxylic group is absent at C-17, a D^17(18)^ double bond occurs, as in triterpenoid **49**; a D^12(13)^ double bond is usually present, as in **22**, while, very rarely, a double bond occurs between C-11 and C-12, as in **21**, or between C-1 and C-2, as in **78**. One compound (**54**) containing a 9(11),12(13)-diene system has also been isolated. A carbonyl group is usually present at C-3, as in **26**, or at C-11, as in **20**; in one compound, **40**, a CO occurs at C-22. An acetal system formed by a β-epoxide bridge between C-25 and C-3 and an α-OH (or, very rarely, an α-alkoxy) group at C-3 is frequently present in oleanane structures, as in **29** or **40**. A few compounds are known to contain a lactone ring formed by a β-oxygen atom at C-13 bonded to a β-CO group at C-17, as in **43**. One example (**34**) of a triterpenoid bearing a β-epoxy ring at C-21/C-22 has been isolated from *L. camara*. A free βOH group usually occurs at C-3, as in **31**, at C-22, as in **26**, and at C-24, as in **28**; very rarely, an OH is present at C-2, as in **78**, or at C-19, as in **25**, at C-7, as in **23**, C-9, as in **64**, and C-12, as in **43**. One 3-*O*-acyl (compound **38**) and one 3-*O*-β-D-glucosyl derivative (**84**) have been isolated. The 22-βOH is usually esterified with an acyl group, e.g., an acetyl, as in **42**, a propanoyl, as in **46**, a butanoyl, as in **50**, an isobutanoyl, as in **51**, an angelyl [(*Z*)-2-methylbut-2-enoyl], as in **48**, and a senecioyl (3-methylbut-2-enoyl) residue, as in **49**; rarer are the esters with (*S*)-2-mehylbutanoic acid, as the triterpenoid **66**, and (*S*)-3-hydroxy-2-methylidenebutanoic acid, as **69**.

Most of these structural characteristics are shared by the ursane triterpenoids due to the close biosynthetic origin of the oleanane and ursane families. A unique ursane triterpenoid is the 3-*O*-β-D-glucosyl derivative **127**, in which stearic acid is esterified to the 4-OH group of a glucosyl moiety.

The flavonoids (Table 4) are represented by 19 flavones (**128–146**), which are mainly apigenin and luteolin derivatives. A semisynthetic derivative (**144**) is included. Three rare *O*-methyl flavonols (**147–149**) and two isoflavones, i.e., 5,7-dihydroxy-6,3′,4′-trimethoxy isoflavone (**150**) and triglycoside **151**, have also been isolated. The two iridoids **152** and **153** (Table 5) are the 1-*O* glucosides of the common aglycones genipin and 4a-OH genipin. The fatty acids **154–162** (Table 6) include common saturated and unsaturated long-chain homologues from C_14_ to C_32_. Finally, the small group of miscellaneous metabolites **163–168** (Table 7) includes the toxic cyanogenic glucoside linamarin (**164**), the common aliphatic alcohols phytol (**165**), and triacontane-1-ol (**167**).

Compounds isolated from *L. camara* and semisynthetic derivatives are listed in the following Table 2, Table 3, Table 4, Table 5, Table 6 and Table 7. The compounds with the same molecular skeleton are grouped and are then listed by increasing molecular formulae. Compounds with the same molecular formula are listed in alphabetic order.

**Table 2:** Steroids isolated from non-volatile fractions of *Lantana camara*.

Nº	Compound Name	Molecular Formula	Molecular Weight	Skeleton Type	Part of the Plant/Solvent	Reference
(**1**)	**Stigmasterol (Stigmasta-5,22-dien-3*β*-ol)**	C_29_H_48_O	412.702	Stigmastane	Leaves/methanol Stems/methanol	[71]
(**2**)	**7-Oxo-*β*-sitosterol** 3*β*-Hydroxy-stigmast-5-en-7-one	C_29_H_48_O_2_	428.701	Stigmastane	Stems/methanol Roots/chloroform	[19,71]
(**3**)	***β*-Sitosterol** Stigmast-5-en-3*β*-ol (Figure 4)	C_29_H_50_O	414.718	Stigmastane	Aerial parts/petroleum ether Aerial parts/96% ethanol Fruits/chloroform Leaves/methanol Stems/95% ethanol, methanol Roots/chloroform Leaves, stems, and roots/ petroleum ether	[19,72,73,74]
(**4**)	***β*-Sitosterol 3-*O*-β-D-glucopyranoside** 3-*O*-β-D-Glucopyranosyl-stigmast-5-en-3β-ol	C_35_H_60_O_6_	576.859	Stigmastane	Aerial parts/methanol Leaves/methanol Stems/95% ethanol, methanol	[15,71,74,75,76,77]
(**5**)	**Campesterol** Campest-5-en-3β-ol	C_28_H_48_O	400.691	Campestane	Leaves/methanol Stems/methanol	[71]
(**6**)	**Yamogenin II** (25*S*)-Spirostan-5-ene-3β,21-diol-3-O-α-L-rhamnopyranosyl-(1″33→2′)-[α-L-rhamnopyranosyl-(1‴→4′)]-β-D-glucopyranoside (Figure 4)	C_45_H_72_O_17_	885.054	Spirostane	Leaves/methanol	[78,79]

**Table 3:** Triterpenoids isolated from non-volatile fractions of *Lantana camara* and semisynthetic derivatives.

Nº	Compound	Molecular Formula	Molecular Weight	Skeleton Type	Part of the Plant/Solvent	Reference
(**7**)	**Alisol A**		490.725	Protostane	Roots/chloroform	[19]
	(8*α*,9*β*,11β,14*β*,23*S*,24*R*)-11,23,24,25-tetrahydroxy-protost-13(17)-en-3-one	C_30_H_50_O_5_				
	(Figure 4)					
(**8**)	**Lantrieuphpene B** (Figure 4)	C_31_H_44_O_5_	496.688	Euphane	Aerial parts/methanol	[80]
(**9**)	**Lantrieuphpene C** (Figure 4)	C_31_H_46_O_5_	498.704	Euphane	Aerial parts/methanol	[80]
(**10**)	**Euphane monolactone A** (Figure 4)	C_32_H_46_O_6_	526.714	Euphane	Leaves/acetonitrile	[72]
(**11**)	**Euphane monolactone B** (Figure 4)	C_32_H_46_O_7_	542.713	Euphane	Leaves/acetonitrile	[72]
(**12**)	**Lantrieuphpene D** (Figure 4)	C_32_H_48_O_6_	528.730	Euphane	Aerial parts/methanol	[80]
(**13**)	**Lantrieuphpene A** (Figure 4)	C_33_H_46_O_7_	554.724	Euphane	Aerial parts/methanol	[80]
(**14**)	**Euphane monolactone C** (Figure 4)	C_40_H_58_O_14_	762.890	Euphane	Leaves/acetonitrile	[72,80]
(**15**)	**Euphane monolactone D** (Figure 4)	C_42_H_60_O_15_	804.927	Euphane	Leaves/acetonitrile	[72]
(**16**)	**Betulonic acid** 3-oxo-lup-20(29)-en-28-oic acid (Figure 5)	C_30_H_46_O_3_	454.695	Lupane	Leaves/methanol Stems/methanol Leaves and stems/petroleum ether	[61,71,79]
(**17**)	**Lantabetulic acid** 3,25-β-epoxy-3*α*-hydroxy-lup-20 (29)-en-28-oic acid (Figure 5)	C_30_H_46_O_4_	470.694	Lupane	Leaves and stems/petroleum ether	[81]
(**18**)	**Betulinic acid** 3*β*-hydroxy-lup-20 (29)-en-28-oic acid	C_30_H_48_O_3_	456.711	Lupane	Aerial parts/methanol Leaves/methanol Stems/methanol Leaves and stems/petroleum ether	[71,72,78,79,82]
(**19**)	**Camaradienone** 3,25-β-epoxy-3α-hydroxy-28-nor-oleana-12,17-dien-11-one (Figure 5)	C_29_H_42_O_3_	438.652	Oleanane	Aerial parts/methanol	[15]
(**20**)	**Lantanoic acid** 3,25-β-epoxy-3α-hydroxy-11-oxo-olean-12-en-28-oic acid (Figure 5)	C_30_H_44_O_5_	484.677	Oleanane	Aerial parts/methanol	[83]
(**21**)	**3*β*-Hydroxy-olean-11-en-28,13-*β*-olide** (Figure 5)	C_30_H_46_O_3_	454.695	Oleanane	HD *	[74]
(**22**)	**Oleanonic acid** 3-Oxo-olean-12-en-28-oic acid (Figure 5)	C_30_H_46_O_3_	454.695	Oleanane	Aerial parts/ethanol, methanol Leaves/ethanol Leaves and stems/methanol, petroleum ether Stems/ethanol, methanol Roots/ethyl acetate, methanol, *n*-hexane–ethyl acetate–methanol (1:1:1)	[15,71,73,74,75,76,77,78,79,81,82,83,84,85,86,87,88]
(**23**)	**Camarin** 7*α*-hydroxy-3-oxo-olean-12-en-28-oic acid (Figure 5)	C_30_H_46_O_4_	470.694	Oleanane	Aerial parts/methanol	[89,90]
(**24**)	**4-*epi*-Hederagonic acid** 24-hydroxy-3-oxo-olean-12-en-28-oic acid (Figure 5)	C_30_H_46_O_4_	470.694	Oleanane	Aerial parts/ethanol Leaves and stems/petroleum ether	[84]
(**25**)	**19*α*-Hydroxy-oleanonic acid** (*S*)-19*α*-hydroxy-3-oxo-olean-12-en-28-oic acid (Figure 5)	C_30_H_46_O_4_	470.694	Oleanane	Aerial parts/methanol	[80]
(**26**)	**22*β*-Hydroxy-oleanonic acid** 22*β*-hydroxy-3-oxo-olean-12-en-28-oic acid (Figure 5)	C_30_H_46_O_4_	470.694	Oleanane	Aerial parts/ethanol HD Leaves/acetonitrile Leaves and stems/petroleum ether	[75,84,91,92,93,94,95,96,97]
(**27**)	**Lantanolic acid** 3,25-β-epoxy-3*α*-hydroxy-olean-12-en-28-oic acid (Figure 5)	C_30_H_46_O_4_	470.694	Oleanane	Aerial parts/methanol HD Leaves/chloroform, methanol, petroleum ether Leaves and stems/petroleum ether Roots/ethanol	[15,75,77,79,82,84,89,90,92, 98]
(**28**)	**22β-Hydroxy-4-*epi*-hederagonic acid** (Figure 5)	C_30_H_46_O_5_	486.693	Oleanane	Aerial parts/ethanol	[84]
(**29**)	**Lantacamaric acid A** 3,25-β-epoxy-3*α*,24-dihydroxy-olean-12-en-28-oic acid (Figure 5)	C_30_H_46_O_5_	486.693	Oleanane	Leaves and stems/methanol	[85]
(**30**)	**Lantaninilic acid** 3,25-β-epoxy-3*α*,22*β*-dihydroxy-olean-12-en-28-oic acid (Figure 5)	C_30_H_46_O_5_	486.693	Oleanane	Aerial parts/methanol HD * Leaves and stems/methanol	[75,82,83,85,89,90]
(**31**)	**Oleanolic acid** 3*β*-hydroxy-olean-12-en-28-oic acid (Figure 6)	C_30_H_48_O_3_	456.711	Oleanane	Aerial parts/ethanol, methanol Leaves/methanol Leaves and stems/petroleum ether Stems/ethanol, methanol Roots/ethanol, ethyl acetate, (52.5% methanol/47.5% ethyl acetate), (60% chloroform/40% methanol), *n*-hexane–ethyl acetate–methanol (1:1:1)	[15,19,74,75,76,77,79,80,82,84,86,87,90,91,98,99,100,101,102]
(**32**)	**22*β*-Hydroxy-oleanolic acid** 3*β*,22*β*-dihydroxy-olean-12-en-28-oic acid (Figure 6)	C_30_H_48_O_4_	472.710	Oleanane	Aerial parts/ethanol HD * Roots/ethanol	[84,95,96]
(**33**)	**Methyl lantanoate** methyl 3,25-β-epoxy-3α-hydroxy-11-oxo-olean-12-en-28-oate (Figure 6)	C_31_H_46_O_5_	498.704	Oleanane	HD *	[83]
(**34**)	21,22-*β*-Epoxy-3*β*-hydroxy-olean-12-en-28-oic acid, isolated as **Methyl 21,22-*β*-epoxy-3*β*-hydroxy-olean-12-en-28-oate** (Figure 6)	C_31_H_48_O_4_	484.721	Oleanane	Roots/*n*-hexane–ethyl acetate–methanol (1:1:1)	[74]
(**35**)	**Methyl 22*β*-hydroxy-oleanonate** methyl 22*β*-hydroxy-3-oxo-olean-12-en-28-oate (Figure 6)	C_31_H_48_O_4_	484.721	Oleanane	HD *	[93,94]
(**36**)	**Methyl lantaninilate** methyl 3,25-β-epoxy-3*α*,22*β*-dihydroxy-olean-12-en-28-oate (Figure 6)	C_31_H_48_O_5_	500.720	Oleanane	HD *	[75]
(**37**)	**22*β*-Acetyloxy-oleanonic acid** 22*β*-acetyloxy-3-oxo-olean-12-en-28-oic acid (Figure 6)	C_32_H_48_O_5_	512.731	Oleanane	HD *	[94]
(**38**)	**Lantanone** 3*β*-acetyloxy-11-oxo-olean-12-en-28-oic acid (Figure 6)	C_32_H_48_O_5_	512.731	Oleanane	Aerial parts/ethanol	[101]
(**39**)	**Methyl 3,25-β-epoxy-3*α*-methoxy-22-oxo-olean-12-en-28-oate** (Figure 6)	C_32_H_48_O_5_	512.731	Oleanane	HD *	[103]
(**40**)	**22β-Acetyloxy-4-*epi*-hederagonic acid** (Figure 6)	C_32_H_48_O_6_	528.730	Oleanane	Aerial parts/ethanol	[104]
(**41**)	**Lancamarinic acid** 22*β*-acetyloxy-3,25-β-epoxy-3*α*-hydroxy-olean-12-en-28-oic acid (Figure 6)	C_32_H_48_O_6_	528.730	Oleanane	Aerial parts/methanol	[105]
(**42**)	**Lancamarolide** 22*β*-acetyloxy-3,25-β-epoxy-3*α*,12*α*-dihydroxyolean-28,13-*β*-olide (Figure 6)	C_32_H_48_O_7_	544.729	Oleanane	Aerial parts/methanol	[82]
(**43**)	**Lancamaric acid** 3,25-β-epoxy-3*α*-ethoxy-olean-12-en-28-oic acid (Figure 6)	C_32_H_50_O_4_	498.748	Oleanane	Aerial parts/methanol	[85]
(**44**)	**Oleanolic acid 3-*O*-acetate** 3*β*-acetyloxy-olean-12-en-28-oic acid (Figure 6)	C_32_H_50_O_4_	498.748	Oleanane	Aerial parts/methanol HD * Roots/*n*-hexane–ethyl acetate–methanol (1:1:1)	[74,77,102,106]
(**45**)	**Methyl 22*β*-acetyloxy-oleanonate** methyl 22*β*-acetyloxy-3-oxo-olean-12-en-28-oate (Figure 6)	C_33_H_50_O_5_	526.758	Oleanane	HD *	[94]
(**46**)	**22*β*-Propanoyloxy-oleanonic acid** 22*β*-propanoyloxy-3-oxo-olean-12-en-28-oic acid (Figure 6)	C_33_H_50_O_5_	526.758	Oleanane	HD *	[94]
(**47**)	**Methyl 22-*O*-acetyl-lantaninilate** methyl 22*β*-acetyloxy-3,25-β-epoxy-3*α*-hydroxy-olean-12-en-28-oate (Figure 6)	C_33_H_50_O_6_	542.757	Oleanane	HD *	[103,107]
(**48**)	**Lantadienone** 22*β*-angelyloxy-3,25-β-epoxy-3α-hydroxy-28-nor-oleana-12,17-dien-11-one (Figure 7)	C_34_H_48_O_5_	536.753	Oleanane	Aerial parts/methanol	[15]
(**49**)	**Lantigdienone** 3,25-β-epoxy-3*α*-hydroxy-11-oxo-22*β*-senecioyloxy-28-nor-olean-12,17-diene (Figure 7)	C_34_H_48_O_5_	536.753	Oleanane	Aerial parts/methanol	[108]
(**50**)	**22*β*-Butanoyloxy-oleanonic acid** 22*β*-butanoyloxy-3-oxo-olean-12-en-28-oic acid (Figure 7)	C_34_H_52_O_5_	540.785	Oleanane	HD *	[94]
(**51**)	**Lantadene D** 22*β*-isobutyryloxy-3-oxo-olean-12-en-28-oic acid (Figure 7)	C_34_H_52_O_5_	540.785	Oleanane	Aerial parts/ethanol HD * Leaves/acetonitrile, methanol, petroleum ether	[19,84,94,97]
(**52**)	**Methyl 22*β*-propanoyloxy-oleanonate** methyl 22*β*-propanoyloxy-3-oxo-olean-12-en-28-oate (Figure 7)	C_34_H_52_O_5_	540.785	Oleanane	HD *	[92]
(**53**)	**24-Hydroxylantadene D** 22*β*-isobutyryloxy-24-hydroxy-3-oxo-olean-12-en-28-oic acid (Figure 7)	C_34_H_52_O_6_	556.784	Oleanane	Aerial parts/ethanol	[84]
**(54)**	**Lantrigloylic acid** 3,25-β-epoxy-3α-hydroxy-22β-senecioyloxy-olea-9 (11),12-dien-28-oic acid (Figure 7)	C_35_H_50_O_6_	566.779	Oleanane	Aerial parts/methanol	[90]
(**55**)	**Camangeloyl acid** 3,25-β-epoxy-3*α*-hydroxy-22*β*-[(*Z*)-2-methyl-2-butenoyloxy]-11-oxo-olean-12-en-28-oic (Figure 7)	C_35_H_50_O_7_	582.778	Oleanane	Aerial parts/methanol	[15,77,83,89,106]
(**56**)	**Camarinin** 3,25-β-epoxy-3*α*-hydroxy-22*β*-(3-methyl-2-butenoyloxy)- 11-oxo-olean-12-en-28-oic (Figure 7)	C_35_H_50_O_7_	582.778	Oleanane	Aerial parts/methanol	[83,84,89,90,107]
(**57**)	**Lantadene A nitrile** 22*β*-angelyloxy-28-ciano-3-oxo-olean-12-ene (Figure 7)	C_35_H_51_NO_3_	533.797	Oleanane	HD *	[93]
(**58**)	**Lantadene A acyl chloride** 28-chloro-22*β*-angelyloxy-3,28-dioxo-olean-12-ene (Figure 7)	C_35_H_51_ClO_4_	571.239	Oleanane	HD *	[93]
(**59**)	**22*β*-Angelyloxy-3-oxo-olean-28,13-*β*-olide** (Figure 7)	C_35_H_52_O_5_	552.796	Oleanane	HD *	[108]
(**60**)	**Lantadene A** **(Rehmannic acid)** 22*β*-angelyloxy-3-oxo-olean-12-en-28-oic acid (Figure 7)	C_35_H_52_O_5_	552.796	Oleanane	Aerial parts/methanol, ethanol Leaves/acetone, acetonitrile, ethanol, ethyl acetate, methanol, petroleum ether, methanol–water (70:30) Leaves and stems/methanol, petroleum ether Roots/*n*-hexane–ethyl acetate–methanol (1:1:1); ethanol Stems/ethanol, methanol	[108]
(**61**)	**Lantadene B** 3-oxo-22β-senecioyloxy- olean-12-en-28-oic acid (Figure 7)	C_35_H_52_O_5_	552.796	Oleanane	Aerial parts/ethanol, dichloromethane, methanol, petroleum ether Leaves/acetonitrile, 96% ethanol, ethyl acetate Leaves/methanol, (70% methanol/30% water) Leaves and stems/petroleum ether Stems/methanol Roots/ethanol	[15,19,79,82,84,91,95,96,98,109,110,111,112,113,114,115,116]
(**62**)	**Camaric acid** 3,25-β-epoxy-3*α*-hydroxy-22*β*-[(*Z*)-2-methyl-2-butenoyloxy]-olean-12-en-28-oic acid (Figure 7)	C_35_H_52_O_6_	568.795	Oleanane	Aerial parts/dichloromethane, methanol Leaves and stems/methanol Roots/*n*-hexane–ethyl acetate–methanol (1:1:1)	[15,74,77,80,82,84,90,98,99,117,118]
(**63**)	**3,25-β-Epoxy-3*α*-hydroxy-22*β*-[(*E*)-2-methyl-2-butenoyloxy]-olean-12-en-28-oic acid** (Figure 7)	C_35_H_52_O_6_	568.795	Oleanane	Aerial parts/ethanol	[84]
(**64**)	**9-Hydroxy-lantadene A** 22*β*-angelyloxy-9-hydroxy-3-oxo-olean-12- en-28-oic acid (Figure 7)	C_35_H_52_O_6_	568.795	Oleanane	Leaves/ethyl acetate, methanol	[119,120]
(**65**)	**24-Hydroxylantadene B** **≡ 24-Hydroxy-22*β*-senecioyloxy-oleanonic acid** 24-hydroxy-3-oxo-22*β*-senecioyloxy-olean-12-en-28-oic acid (Figure 7)	C_35_H_52_O_6_	568.795	Oleanane	Aerial parts/ethanol Leaves/ethyl acetate Leaves and stems/methanol	[84,99,116]
(**66**)	**24-Hydroxy lantadene X** 24-hydroxy-3-oxo-22*β*-[(*E*)-2-methylbut-2-enoyloxy]- olean-12-en-28-oic acid (Figure 7)	C_35_H_52_O_6_	568.795	Oleanane	Aerial parts/ethanol	[84]
(**67**)	**Icterogenin** **≡ 24-Hydroxy-lantadene A** **≡ 24-Hydroxy-22*β*-angelyloxy-oleanonic acid** 24-hydroxy-3-oxo-22*β*-[(*Z*)-2-methylbut-2-enoyloxy]- olean-12-en-28-oic acid (Figure 7)	C_35_H_52_O_6_	568.795	Oleanane	Aerial parts/ethanol, methanol Leaves/acetone, ethanol Leaves/ethyl acetate, methanol Leaves and stems/methanol, petroleum ether	[19,79,80,81,82,84,99,109,116,118,121]
(**68**)	**Lantanilic acid** 3*β*,25-β-epoxy-3*α*-hydroxy-22*β*-senecioyloxy-olean-12-en-28-oic acid (Figure 7)	C_35_H_52_O_6_	568.795	Oleanane	Aerial parts/dichloromethane, ethanol, methanol Leaves/ethanol, ethyl acetate, methanol, petroleum ether Leaves and stems/methanol Roots/chloroform	[15,19,72,77,79,81,84,85,98,99,100,109,116,122,123]
(**69**)	**Camarolic acid** 3,25-β-epoxy-3*α*-hydroxy-22*β*-[(*S*)-3-hydroxy-2-methylidenebutanoyloxy] olean- 12-en-28-oic acid (Figure 7)	C_35_H_52_O_7_	584.794	Oleanane	Aerial parts/methanol	[82,90]
(**70**)	**Lantacamaric acid B** 3,25-β-epoxy-3*α*,24-dihydroxy-22*β*-senecioyloxy-olean-12-en-28-oic acid (Figure 7)	C_35_H_52_O_7_	584.794	Oleanane	Leaves and stems/methanol	[85]
(**71**)	**Lantadene A amide** 28-amino-22*β*-angelyloxy-3,28-dioxo-olean-12-ene (Figure 7)	C_35_H_53_NO_4_	551.812	Oleanane	HD *	[93]
(**72**)	**Lantadene C** 22*β*-[(*S*)-2-methylbutanoyloxy]-3-oxo-olean-12-en-28-oic acid (Figure 7)	C_35_H_54_O_5_	554.812	Oleanane	HD * Leaves/acetonitrile, ethyl acetate, methanol Leaves and stems/petroleum ether	[91,94,109, 116,124]
(**73**)	**Methyl 22*β*-butanoyloxy-oleanonate** methyl 22*β*-butanoyloxy-3-oxo-olean-12-en-28-oate (Figure 7)	C_35_H_54_O_5_	554.812	Oleanane	HD *	[94]
(**74**)	**Methyl 22*β*-isobutyryloxy-oleanonate** methyl 22*β*-isobutyryloxy-3-oxo-olean-12-en-28-oate (Figure 7)	C_35_H_54_O_5_	554.812	Oleanane	HD *	[94]
(**75**)	**Reduced lantadene A** 22*β*-angelyloxy-3*β*-hydroxy-olean-12-en-28-oic acid (Figure 8)	C_35_H_54_O_5_	554.812	Oleanane	Aerial parts/ethanol HD * Leaves/methanol, acetonitrile Roots/ethanol	[84,91,95]
(**76**)	**Reduced lantadene B** 3*β*-hydroxy-22*β*-senecioyloxy-olean-12-en-28-oic acid (Figure 8)	C_35_H_54_O_5_	554.812	Oleanane	Aerial parts/ethanol HD * Leaves/acetonitrile, methanol Roots/ethanol	[84,91,95]
(**77**)	**Reduced lantadene C** 3*β*-hydroxy-22*β*-[2-methylbutanoyloxy]-olean-12-en-28-oic acid (Figure 8)	C_35_H_56_O_5_	554.812	Oleanane	Aerial parts/ethanol	[84]
(**78**)	**Methyl 22*β*-angelyloxy-2-hydroxy-3-oxo-olean-1,12-diene-** **28-oate** (Figure 8)	C_36_H_52_O_6_	580.806	Oleanane	HD *	[124]
(**79**)	**Lancamarinin** methyl 3,25-β-epoxy-3*α*-hydroxy-11-oxo-22*β*-senecioyloxy-olean-12-en- 28-oate (Figure 8)	C_36_H_52_O_7_	596.805	Oleanane	HD * Aerial parts/methanol	[105]
(**80**)	**Methyl camangeloylate** methyl 3,25-β-epoxy-3*α*-hydroxy-22*β*-[(*Z*)-2′-methyl-2′-butenoyloxy]-11-oxo-olean-12-en-28-oate (Figure 8)	C_36_H_52_O_7_	596.805	Oleanane	HD *	[77]
(**81**)	**Lantadene A** **methyl ester** methyl 22*β*-angelyloxy-3-oxo-olean-12-en-28-oate (Figure 8)	C_36_H_54_O_5_	566.823	Oleanane	HD * Leaves and stems/petroleum ether	[93,124]
(**82**)	**Methyl 22-*β*-angelyloxy-lantanolate** methyl 22*β*-angelyloxy-3,25-β-epoxy-3*α*-hydroxy-olean-12-en-28-oate (Figure 8)	C_36_H_54_O_6_	582.822	Oleanane	HD *	[82]
(**83**)	**Methyl camarolate** methyl 3,25-β-epoxy-3*α*-hydroxy-22*β*-[(*S*)-3-hydroxy-2-methylidenebutanoyloxy] olean-12-en-28-oate (Figure 8)	C_36_H_54_O_7_	598.821	Oleanane	HD *	[90]
(**84**)	**3-*O-β*-D**-**Glucosyl oleanolic acid** 3-*O*-β-D-glucopyranosyloxy-olean-12-en-28-oic acid (Figure 8)	C_36_H_58_O_8_	618.852	Oleanane	Leaves/methanol	[125,126]
(**85**)	**22*β*-Benzoyloxy-oleanonic acid** 22*β*-benzoyloxy-3-oxo-olean-12-en-28-oic acid (Figure 8)	C_37_H_50_O_5_	574.802	Oleanane	HD *	[94]
(**86**)	**Methyl 22*β*-benzoyloxy-oleanonate** methyl 22*β*-benzoyloxy-3-oxo-olean-12-en-28-oate (Figure 8)	C_38_H_52_O_5_	588.829	Oleanane	HD *	[94]
(**87**)	**3*β*-(2-Acetyloxybenzoyloxy)-22*β*-hydroxy-olean-12-en-28-oic acid** (Figure 8)	C_39_H_54_O_7_	634.854	Oleanane	HD *	[96]
(**88**)	**3*β*-[(*R*,*S*)-2-(4-Isobutylphenyl)propanoyloxy]-22*β*-hydroxy-olean-12-en-28-oic acid** (Figure 8)	C_43_H_64_O_5_	660.980	Oleanane	HD *	[96]
(**89**)	**3*β*-{2-[2-(2,6-Dichlorophenylamino)phenyl]acetyloxy}-22*β*-hydroxy-olean-12-en-28-oic acid** (Figure 8)	C_44_H_57_Cl_2_NO_5_	750.842	Oleanane	HD *	[96]
(**90**)	**3*β*-[(+)-(*S*)-2-(6-Methoxy-2-naphthyl)propanoyloxy]-22*β*-hydroxy-olean-12-en-28-oic acid** (Figure 8)	C_44_H_60_O_6_	684.958	Oleanane	HD *	[96]
(**91**)	**3*β*-[(*R*,*S*)-2-(3-Benzoylphenyl)propanoyloxy]-22β-hydroxy-olean-12-en-28-oic acid** (Figure 9)	C_46_H_60_O_6_	708.980	Oleanane	HD *	[96]
(**92**)	**3*β*,22*β*-Di-(2-acetyloxybenzoyloxy)-olean-12-en-28-oic acid** (Figure 9)	C_48_H_60_O_10_	796.998	Oleanane	HD *	[96]
(**93**)	**3*β*,22*β*-Di-[(*R*.*S*)-2-(4-isobutylphenyl)propanoyloxy]-olean-12-en-28-oic acid** (Figure 9)	C_56_H_80_O_6_	849.250	Oleanane	HD *	[96]
(**94**)	**3*β*,22*β*-Di-{2-[2-(2,6-dichlorophenylamino)phenyl]acetyloxy}-olean-12-en-28-oic acid** (Figure 9)	C_58_H_66_Cl_4_N_2_ O_6_	1028.974	Oleanane	HD *	[96]
(**95**)	**3*β*,22*β*-Di-[(+)-(*S*)-2-(6-methoxy-2-naphthyl)propanoyloxy]-olean-12-en-28-oic acid** (Figure 9)	C_58_H_72_O_8_	897.206	Oleanane	HD *	[96]
(**96**)	**3*β*,22*β*-Di-[(*R*,*S*)-2-(3-benzoylphenyl)propanoyloxy]-olean-12-en-28-oic acid** (Figure 9)	C_62_H_72_O_8_	945.250	Oleanane	HD *	[96]
(**97**)	**Camarolide** 3-oxo-urs-11-en-28,13-*β*-olide (Figure 10)	C_30_H_44_O_3_	452.679	Ursane	Aerial parts/methanol	[85]
(**98**)	**3,24-Dioxo-urs-12-en-28-oic acid** (Figure 10)	C_30_H_44_O_4_	468.678	Ursane	Leaves/solvent not reported	[127]
(**99**)	**Camaranoic acid** 3,25-β-epoxy-3*α*-hydroxy-11-oxo-urs-12-en-28-oic acid (Figure 10)	C_30_H_44_O_5_	484.677	Ursane	Aerial parts/methanol	[81,83,118]
(**100**)	**Ursonic acid** 3-oxo-urs-12-en-28-oic acid (Figure 10)	C_30_H_46_O_3_	454.695	Ursane	Aerial parts/methanol Leaves and stems/petroleum ether	[81,83,84,85]
(**101**)	**11*α*-Hydroxy-3-oxo-urs-12-en-28-oic acid** (Figure 10)	C_30_H_46_O_4_	470.694	Ursane	Aerial parts/methanol	[82]
(**102**)	**Lantic acid** 3,25-β-epoxy-3*α*-hydroxy-urs-12-en-28-oic acid (Figure 10)	C_30_H_46_O_4_	470.694	Ursane	Aerial parts/methanol Leaves/chloroform Leaves and stems/dichloromethane–methanol (1:1), petroleum ether	[75,83,98]
**(103)**	**11-Oxo-*β*-boswellic acid** 3*α*-hydroxy-11-oxo-urs-12-en-24-oic acid (Figure 10)	C_30_H_46_O_4_	470.694	Ursane	Leaves/ethyl acetate	[120]
(**104**)	**Pomonic acid** 19*α*-hydroxy-3-oxo-urs-12-en-28-oic acid (Figure 10)	C_30_H_46_O_4_	470.694	Ursane	Aerial parts/ethanol, methanol Roots/ *n*-hexane–ethyl acetate–methanol (1:1:1)	[74,77,80,84]
(**105**)	**Lantoic acid** 3,25-β-epoxy-3*α*,22β-dihydroxy-urs-12-en-28-oic acid (Figure 10)	C_30_H_46_O_5_	486.693	Ursane	Aerial parts/methanol Leaves/petroleum ether	[81,84,90,118]
(**106**)	**Ursolic acid** 3*β*-hydroxy-urs-12-en-28-oic acid; urs-12-en-3β-ol-28-oic acid (Figure 10)	C_30_H_48_O_3_	456.711	Ursane	Aerial parts/methanol Leaves/methanol	[19,79,89,90,121,125]
(**107**)	**Pomolic acid** 3*β*,19*α*-dihydroxy-urs-12-en-28-oic acid (Figure 10)	C_30_H_48_O_4_	472.710	Ursane	Aerial parts/methanol Stems/methanol Roots/chloroform, ethanol	[84,89,90,98]
(**108**)	***α*-Amyrin** urs-12-en-3*β*-ol	C_30_H_50_O	426.729	Ursane	Aerial parts/96% ethanol, petroleum ether	[73,74]
(**109**)	**3*β*,19*α*-Dihydroxy-ursan-28-oic acid** (Figure 10)	C_30_H_50_O_4_	474.726	Ursane	Roots/ *n*-hexane–ethyl acetate–methanol (1:1:1)	[74]
(**110**)	**Methyl camaranoate** methyl 3,25-β-epoxy-3*α*-hydroxy-11-oxo-urs-12-en-28-oate (Figure 10)	C_31_H_46_O_5_	498.704	Ursane	HD *	[83]
(**111**)	**Ursoxy acid** 3,25-β-epoxy-3*α*-methoxy-urs-12-en-28-oic acid (Figure 10)	C_31_H_48_O_4_	484.721	Ursane	Aerial parts/methanol	[106]
(**112**)	**Methyl 25-hydroxy-3-deoxy-ursen-12-en-28-oate** (Figure 10)	C_31_H_50_O_3_	470.738	Ursane	HD *	[128]
(**113**)	**Methyl 3*β*,19*α*-dihydroxy ursan-28-oate** (Figure 10)	C_31_H_52_O_4_	488.753	Ursane	HD *	[74]
(**114**)	**Camarinic acid** 22*β*-acetyloxy-3,25-β-epoxy-3*α*-hydroxy-12-ursen-28-oic acid (Figure 10)	C_32_H_48_O_6_	528.730	Ursane	Aerial parts/methanol Leaves/chloroform Leaves and stems/dichloromethane–methanol (1:1)	[16,83,98, 109]
(**115**)	**Methyl ursoxylate** methyl 3,25-β-epoxy-3*α*-methoxy-urs-12-en-28-oate (Figure 10)	C_32_H_50_O_4_	498.748	Ursane	Aerial parts/methanol HD *	[106]
(**116**)	**Ursethoxy acid** 3,25-β-epoxy-3*α*-ethoxy-urs-12-en-28-oic acid (Figure 10)	C_32_H_50_O_4_	498.748	Ursane	Aerial parts/methanol	[129]
(**117**)	**Methyl camaralate** methyl 22*β*-acetoxy-3,25-β-epoxy-3*α*-hydroxy-urs-12-en-28-oate (Figure 10)	C_33_H_50_O_6_	542.757	Ursane	Aerial parts/methanol HD *	[77,98]
(**118**)	**Methyl ursethoxylate** methyl 3,25-β-epoxy-3α-ethoxy-urs-12-en-28-oate (Figure 10)	C_33_H_52_O_4_	512.775	Ursane	Aerial parts/methanol	[129]
(**119**)	**Lantacin** 3*β*,19*α*-dihydroxy-22*β*-senecioyloxy-urs-12-en-28-oic acid (Figure 10)	C_35_H_54_O_6_	570.811	Ursane	Aerial parts/methanol	[84,89,118]
(**120**)	**Lantaiursolic acid** 3*β*-isovaleroyloxy-19*α*-hydroxy-urs-12-en-28-oic acid (Figure 10)	C_35_H_56_O_5_	556.828	Ursane	Roots/ethanol	[118]
(**121**)	**Camaracinic acid** 22*β*-angelyloxy-3,25-β-epoxy-3*α*-methoxy-12-ursen-28-oic acid (Figure 11)	C_36_H_54_O_6_	582.822	Ursane	Aerial parts/methanol	[82]
(**122**)	**Camaryolic acid** 3,25-β-epoxy-3*α*-methoxy-22*β*-senecioyloxy-urs-12-en-28-oic acid (Figure 11)	C_36_H_54_O_6_	582.822	Ursane	Aerial parts/methanol	[77,82]
(**123**)	**Methyl lantacinate** methyl 3*β*,19*α*-dihydroxy-22*β*-senecioyloxy- urs-12-en-28-oate (Figure 11)	C_36_H_56_O_6_	584.838	Ursane	HD *	[84]
(**124**)	**Methyl camaracinate** methyl 22*β*-angelyloxy-3,25-β-epoxy-3*α*-methoxy-12-ursen-28-oate (Figure 11)	C_37_H_56_O_6_	596.849	Ursane	HD *	[82]
(**125**)	**Methyl camaryolate** methyl 3,25-β-epoxy-3*α*-methoxy-22*β*-senecioyloxy-urs-12-en-28-oate (Figure 11)	C_37_H_56_O_6_	596.849	Ursane	HD *	[82]
(**126**)	**Ursangilic acid** 22β-angelyloxy- 3,25-β-epoxy-3*α*-ethoxy-urs-12-en-28-oic acid (Figure 8)	C_37_H_56_O_6_	596.849	Ursane	Aerial parts/methanol	[106]
(**127**)	**Urs-12-en-3β-ol-28-oic acid 3-*O*-β-D-glucopyranosyl-4′- octadecanoate** (Figure 11)	C_54_H_92_O_9_	885.321	Ursane	Leaves/methanol	[125,126]

* HD: semisynthetic derivative.

**Table 4:** Flavonoids isolated from non-volatile fractions of *Lantana camara* and semisynthetic derivatives.

N.º	Compound	Molecular Formula	Molecular Weight	Flavonoid Type	Part of the Plant/Solvent	Reference
(**128**)	**Hispidulin** 4′,5,7-trihydroxy-6-methoxyflavone (Figure 12)	C_16_H_12_O_6_	300.266	Flavone	Leaves/ethanol Stems/methanol	[63,79,84]
(**129**)	**Pectolinarigenin** 5,7-dihydroxy-4′,6-dimethoxyflavone (Figure 12)	C_17_H_14_O_6_	314.293	Flavone	Leaves/methanol	[19,79,84,130]
(**130**)	**Tricin** 4′,5,7-trihydroxy-3′,5′-dimethoxyflavone (Figure 12)	C_17_H_14_O_7_	330.292	Flavone	Leaves/methanol	[79]
(**131**)	**Apigenin 7-*O*-*β*-D-galacturonide** 7-*O*-*β*-D-galacturonyl-4′,5,7-trihydroxyflavone (Figure 12)	C_21_H_18_O_11_	446.364	Flavone	Flowers/methanol–water (70:30)	[131]
(**132**)	**Anthemoside** **Apigenin 7-*O*-*β*-D-glucopyranoside** 7-*O*-*β*-D-glucopyranosyl- 4′,5,7-trihydroxyflavone (Figure 12)	C_21_H_20_O_10_	432.381	Flavone	Flowers/methanol–water (70:30)	[132]
(**133**)	**Isovitexin** 6-C-*β*-D-glucopyranosyl-4′,5,7-trihydroxyflavone (Figure 12)	C_21_H_20_O_10_	432.381	Flavone	Flowers/ methanol–water (70:30)	[132]
(**134**)	**Vitexin** 8-C-*β*-D-glucopyranosyl-4′,5,7-trihydroxyflavone (Figure 12)	C_21_H_20_O_10_	432.381	Flavone	Flowers/ methanol–water (70:30)	[132,133]
(**135**)	**Juncein** Luteolin 4′-*O*-*β*-D-glucopyranoside 4′-O-*β*-D-glucopyranosyl-3′,4′,5,7-tetrahydroxyflavone (Figure 12)	C_21_H_20_O_11_	448.380	Flavone	Flowers/methanol–water (70:30)	[132]
(**136**)	**Luteolin 7-*O*-*β*-D-galactopyranoside** 7-*O*-*β*-D-galactopyranosyl-3′,4′,5,7-tetrahydroxyflavone (Figure 12)	C_21_H_20_O_11_	448.380	Flavone	Flowers/methanol–water (70:30)	[132]
(**137**)	**Luteoloside** Luteolin 7-*O*-β-glucopyranoside 7-O-*β*-D-glucopyranosyl-3′,4′,5,7-tetrahydroxyflavone (Figure 12)	C_21_H_20_O_11_	448.380	Flavone	Flowers/methanol–water (70:30)	[132]
(**138**)	**6-Methoxy scutellarin** 7-O-*β*-glucuronyl-4′,5,7-trihydroxy-6-methoxyflavone (Figure 12)	C_22_H_20_O_12_	476.390	Flavone	Leaves and stems/methanol	[85]
(**139**)	**Linaroside** 7-*O*-*β*-D-glucopyranosyl-5,7-dihydroxy-4′,6-dimethoxyflavone (Figure 13)	C_23_H_24_O_11_	476.434	Flavone	Aerial parts/ methanol Leaves/ methanol	[16,124,134]
(**140**)	**Lantanoside** 7-*O*-(6-*O*-acetyl-*β*-D-glucopyranosyl)-5,7-dihydroxy-4′,6-dimethoxyflavone (Figure 13)	C_25_H_26_O_12_	518.471	Flavone	Aerial parts/methanol	[16,134]
(**141**)	**Apigenin 7-*O*-*β*-D-galacturonyl-** **(2″→1‴)-*O*-*β*-D-galacturonide** (Figure 13)	C_27_H_26_O_17_	622.488	Flavone	Flowers/methanol–water (70:30)	[132]
(**142**)	**Luteolin 7-*O*-*β*-D-galacturonyl-(2″→1‴)-O-*β*-D-galacturonide** (Figure 13)	C_27_H_26_O_18_	638.487	Flavone	Flowers/methanol–water (70:30)	[132]
(**143**)	**Luteolin 7-O-*β*-D-glucuronyl-(2″→1‴)-O-*β*-D-glucuronide** (Figure 13)	C_27_H_26_O_18_	638.487	Flavone	Flowers/methanol–water (70:30)	[132]
(**144**)	**Acetyl lantanoside** 7-*O*-(2,6-*O*-diacetyl-*β*-D-glucopyranosyl)-5,7-dihydroxy-4′,6-dimethoxyflavone (Figure 13)	C_27_H_28_O_13_	560.508	Flavone	HD *	[16,134]
(**145**)	**Acacetin-7-*O*-*β*-D-rutinoside** 7-*O*-*β*-D-rutinosyl-5,7-dihydroxy- 4′-methoxyflavone (Figure 13)	C_28_H_32_O_14_	592.550	Flavone	Leaves/methanol	[79]
(**146**)	**Pectolinarin** 7-*O*-*β*-D-rutinosyl -5,7-dihydroxy-4′,6-dimethoxyflavone (Figure 13)	C_29_H_34_O_15_	622.576	Flavone	Aerial parts/ ethanol Leaves/ ethanol, methanol	[19,84,133]
(**147**)	**3,7-*O*-Dimethylquercetin** 3′,4′,5-trihydroxy-3,7-dimethoxyflavone (Figure 13)	C_17_H_14_O_7_	330.292	Flavonol	Leaves/acetone	[127]
(**148**)	**3,5,7,8-Tetrahydroxy-3′,6-dimethoxyflavone** (Figure 13)	C_17_H_14_O_8_	346.291	Flavonol	Leaves/methanol	[79]
(**149**)	**6-Methoxykaempferol-7-*O*-β-D-glucoside** 7-O-*β*-D-glucopyranosyl-3,4′,5,7-tetrahydroxy-6-methoxyflavone (Figure 13)	C_22_H_22_O_12_	478.406	Flavonol	Flowers/95% methanol	[135]
(**150**)	**5,7-Dihydroxy-6,3′,4′-trimethoxy isoflavone** (Figure 13)	C_18_H_15_O_7_	343.311	Isoflavone	Leaves/methanol	[79]
(**151**)	**Gautin** 5,7-dihydroxy-6,3′,4′-trimethoxy isoflavone-5-*O*-*α*-L-rhamnopyranosyl-7-O-*β*-D-arabinopyranosyl-(1‴→4″)-O-*β*- D-xylopyranoside (Figure 13)	C_34_H_42_O_19_	754.691	Isoflavone	Leaves/methanol	[79]

* HD: semisynthetic derivative.

**Table 5:** Iridoid glucosides isolated from non-volatile fractions of *Lantana camara* L.

N.º	Compound	Molecular Formula	Molecular Weight	Part of the Plant/Solvent	Reference
(**152**)	**Geniposide** methyl (1*S*,4a*S*,7a*S*)-7-(hydroxymethyl)-1-[(2*S*,3*R*,4*S*,5*S*,6*R*)-3,4,5-trihydroxy-6-(hydroxymethyl) oxan-2-yl] oxy-1,4a,5,7a-tetrahydrocyclopenta[c]pyran-4-carboxylate (Figure 14)	C_17_H_24_O_10_	388.369	Leaves and stems/ methanol Roots/ ethanol	[91]
(**153**)	**Theviridoside** methyl (1*S*,4a*R*,7a*R*)-4a-hydroxy-7-(hydroxymethyl)-1-[(2*S*,3*R*,4*S*,5*S*,6*R*)-3,4,5-trihydroxy-6-(hydroxymethyl) oxan-2-yl] oxy-5,7a-dihydro-1*H*-cyclopenta[c]pyran-4-carboxylate (Figure 14)	C_17_H_24_O_11_	404.368	Aerial parts and roots/ ethanol Leaves and stems/ methanol Roots/ methanol	[91]

**Table 6:** Fatty acids isolated from non-volatile fractions of *Lantana camara* L.

N.º	Compound	Molecular Formula	Molecular Weight	Part of the Plant/Solvent	References
(**154**)	**Myristic acid** tetradecanoic acid	C_14_H_28_O_2_	228.376	Aerial parts/petroleum ether	[73]
(**155**)	**Palmitic acid** hexadecanoic acid	C_16_H_32_O_2_	256.430	Aerial parts/methanol/petroleum ether Stems/ethanol	[73,77,82]
(**156**)	**Linoleic acid** (*9Z*,*12Z*)-octadeca-9,12-dienoic acid	C_18_H_32_O_2_	280.452	Aerial parts/petroleum ether	[73]
(**157**)	**Oleic acid** (9*Z*)-octadec-9-enoic acid	C_18_H_34_O_2_	282.468	Aerial parts/petroleum ether	[73]
(**158**)	**Stearic acid** octadecanoic acid	C_18_H_36_O_2_	284.484	Aerial parts/methanol Stems/ethanol	[77,82]
(**159**)	**Arachidic acid** eicosanoid acid (Figure 14)	C_20_H_40_O_2_	312.538	Aerial parts/petroleum ether	[73]
(**160**)	**Behenic acid** docosanoic acid (Figure 14)	C_22_H_44_O_2_	340.592	Aerial parts/methanol	[77,82]
(**161**)	**Lignoceric acid** tetracosanoic acid (Figure 14)	C_24_H_48_O_2_	368.646	Aerial parts/methanol	[106]
(**162**)	**Lacceroic acid** dotriacontanoic acid (Figure 14)	C_32_H_64_O_2_	480.862	Aerial parts/methanol	[106]

**Table 7:** Other compounds isolated from non-volatile fractions of *Lantana camara* L.

N.º	Compound	Molecular Formula	Molecular Weight	Part of the Plant/Solvent	References
(**163**)	**Ethyl-*β*-D-galactopyranoside** (Figure 14)	C_8_H_16_O_6_	208.210	Stems/ ethanol	[15]
(**164**)	**Linamarin** 2-(*β*-D-Glucopyranosyloxy)-2-methylpropanenitrile (Figure 14)	C_10_H_17_NO_6_	247.247	Leaves and stems/ methanol	[99]
(**165**)	**Phytol** 3,7,11,15-tetramethyl-2-hexadecen-1-ol (Figure 14)	C_20_H_40_O	296.539	Leaves and stems/ petroleum ether	[75]
(**166**)	**Di-(2-ethylhexyl) phthalate** (Figure 14)	C_24_H_38_O_4_	390.564	Fruits/ chloroform	[73]
(**167**)	**Triacontan-1-ol** (Figure 14)	C_30_H_62_O	438.825	Aerial parts/ petroleum ether	[73]
(**168**)	**Trilinolein** Glyceryl trilinoleate (Figure 14))	C_56_H_96_O_6_	865.37	Fruits/ chloroform	[73]

The chemical structures of steroids, triterpenes, flavonoids, iridoid glycosides, fatty acids, and miscellaneous compounds isolated from *L. camara* or obtained by semisynthesis are depicted in Figure 4, Figure 5, Figure 6, Figure 7, Figure 8, Figure 9, Figure 10, Figure 11, Figure 12, Figure 13 and Figure 14. The structures of very well-known compounds have been omitted.

**Figure 4:**
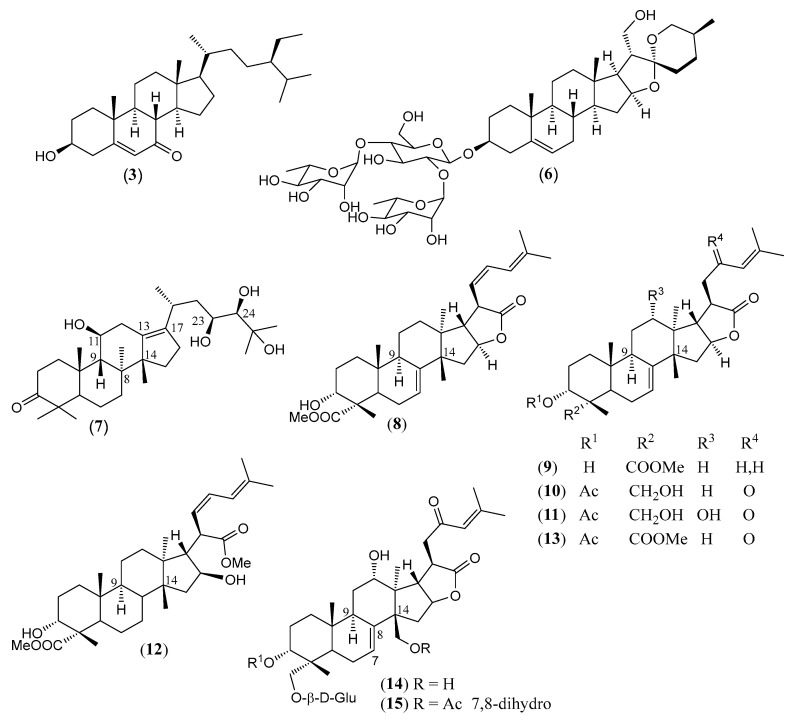
Structures of compounds **3**, and **6**–**15**.

**Figure 5:**
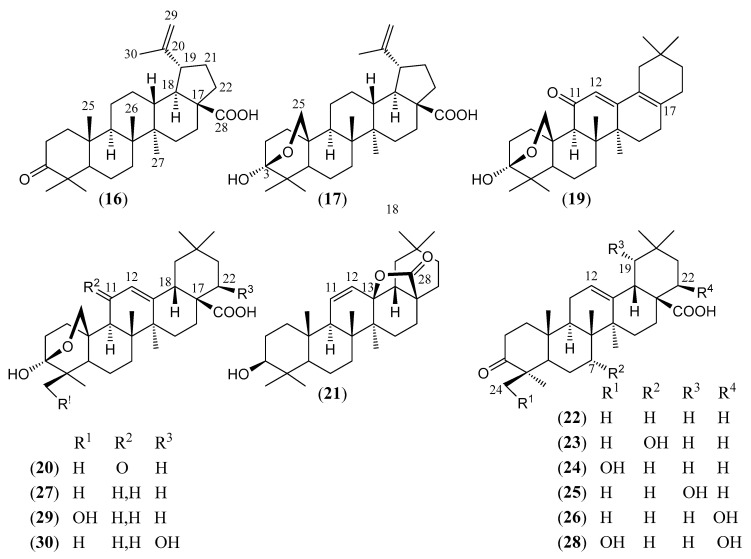
Structures of compounds **16**, **17**, and **19**–**30**.

**Figure 6:**
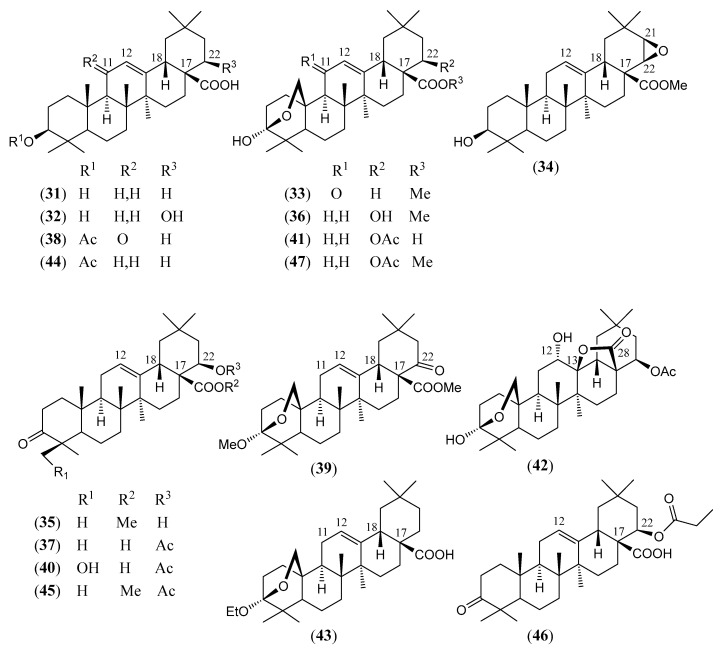
Structures of compounds **31**–**47**.

**Figure 7:**
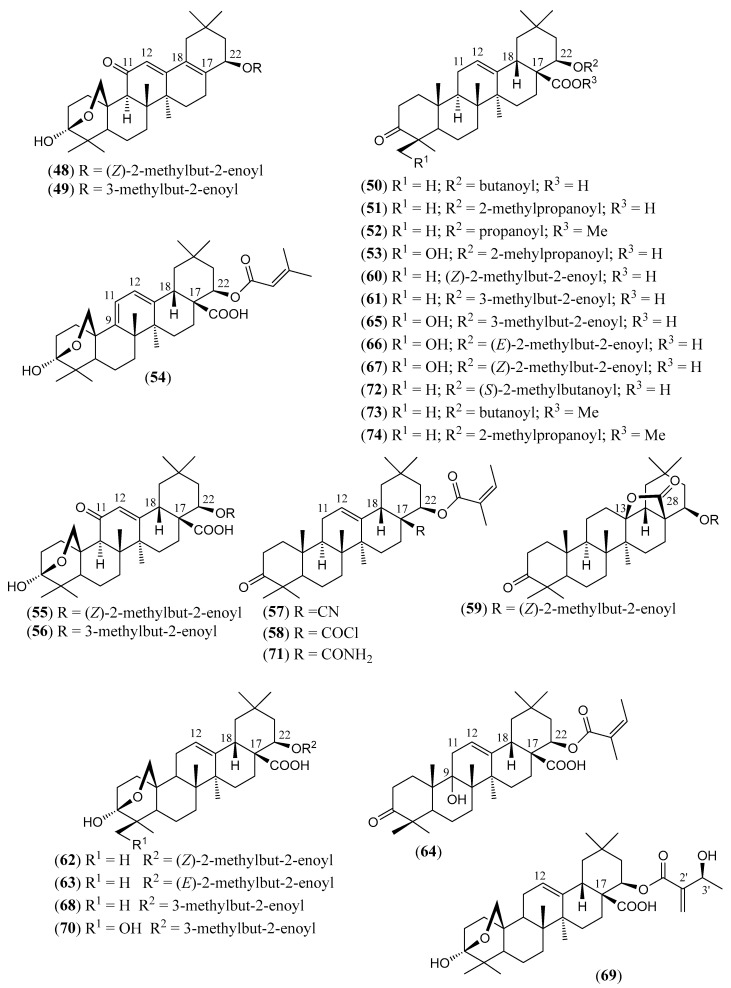
Structures of compounds **48**–**74**.

**Figure 8:**
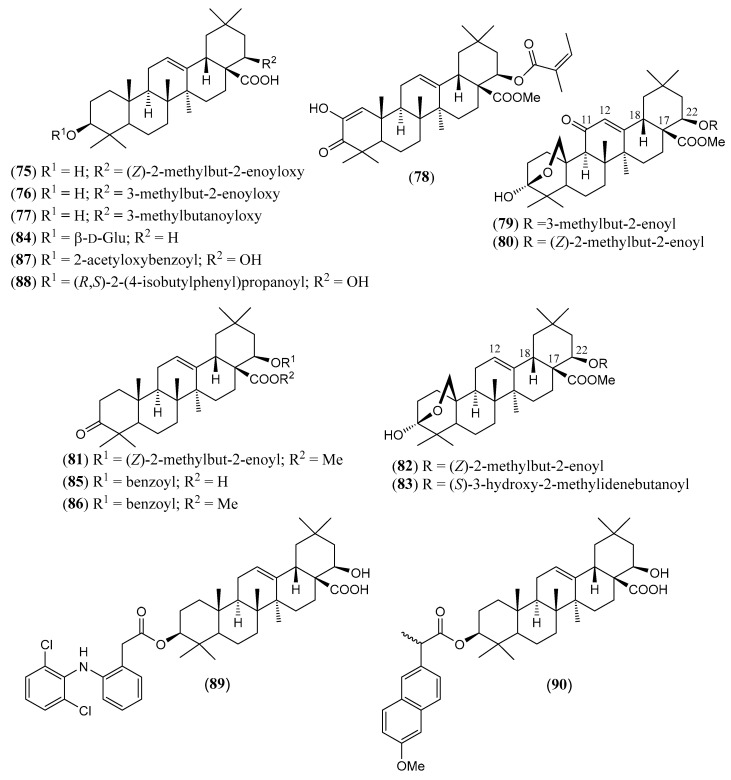
Structures of compounds **75**–**90**.

**Figure 9:**
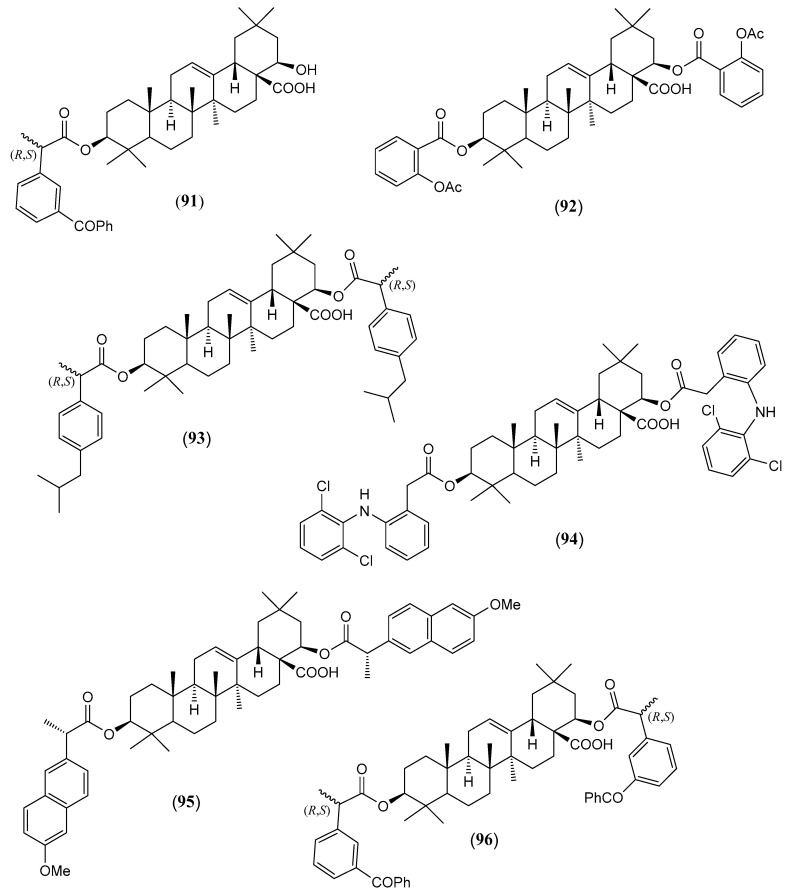
Structures of compounds **91**–**96**.

**Figure 10:**
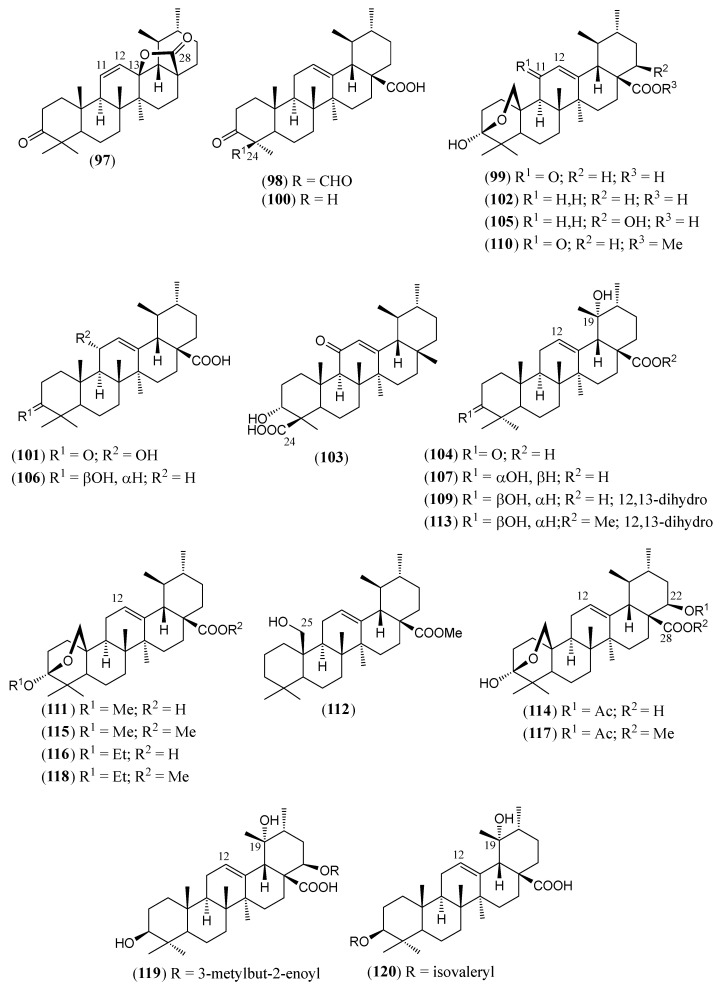
Structures of compounds **97–107** and **109**–**120**.

**Figure 11:**
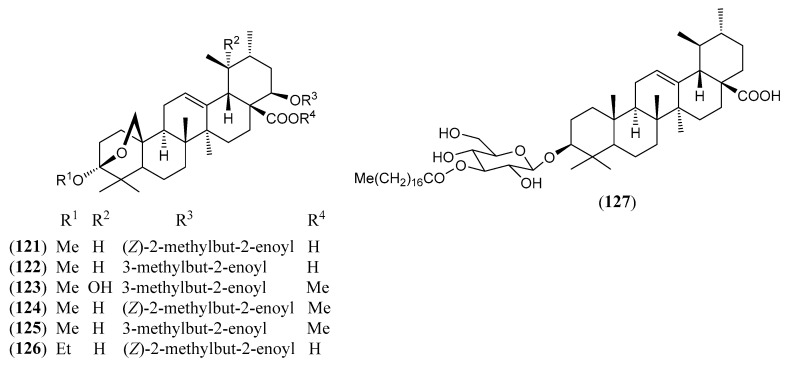
Structures of compounds **121**–**127**.

**Figure 12:**
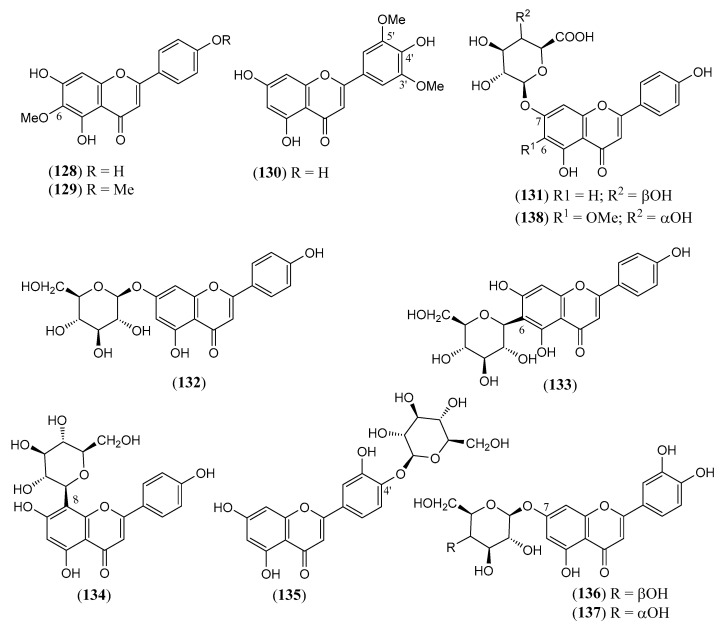
Structures of compounds **128**–**138**.

**Figure 13:**
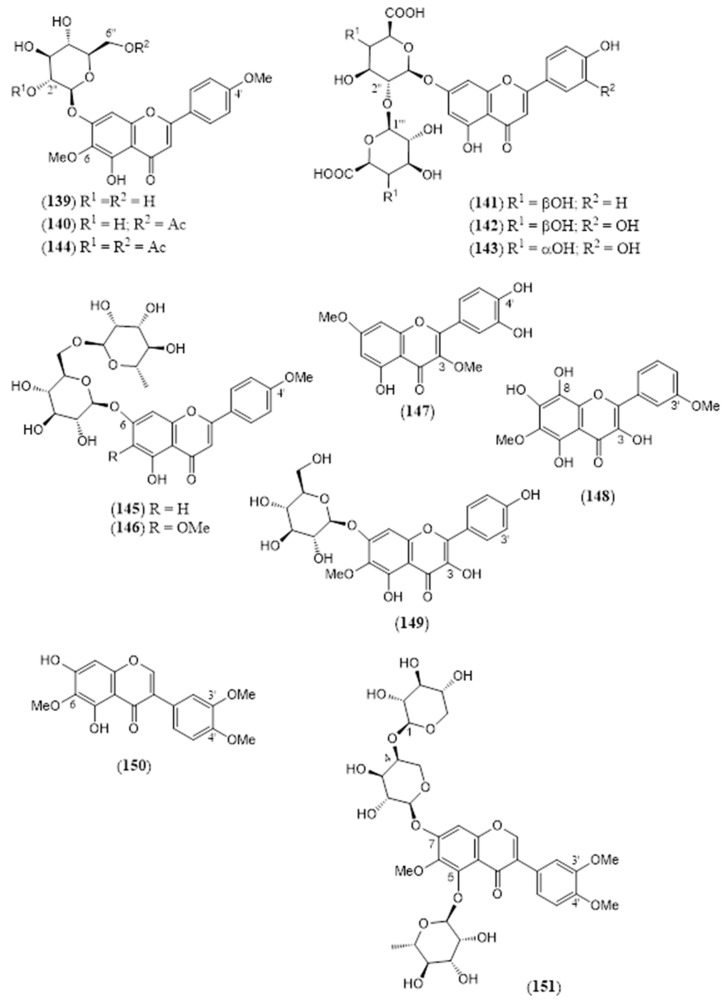
Structures of compounds **139**–**151**.

**Figure 14:**
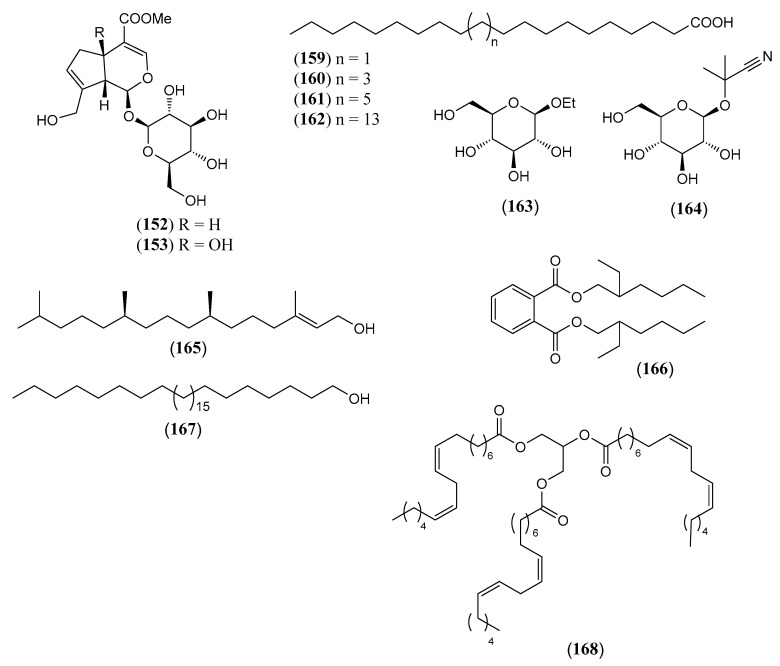
Structures of compounds **152**, **153**, and **159**–**168**.

### 3.3. Biological Activities

A great variety of biological effects exerted by several extracts of different parts of *L. camara* have been tested in vitro and, more rarely, also in vivo (Table 8). The most relevant biological properties included significant anti-inflammatory and analgesic effects of a leaf aqueous extract [136]; a moderate antibacterial activity of leaf alcoholic and aqueous extracts against *Escherichia coli*, *Proteus vulgaris*, and *Vibrio cholerae* [121], and against *Bacillus subtilis*, *Klebsiella pneumoniae*, *Staphylococcus aureus*, and *Pseudomonas aeruginosa* [137]; the inhibitory properties of a 70% aqueous ethanolic flower extract against the growth of the *Mycobacterium tuberculosis* H37RV strain [138]; the antidiabetic effects of an aqueous leaf extract [125]; the moderate antidiarrheal effects of an ethanol leaf extract [100]; the significant antioxidant properties of methanol leaf and flower extracts [139]; the high in vitro antiparasitic activity of dichloromethane and methanol extracts of the leaves/aerial parts against *Leishmania amazonensis* promastigotes [64,67,109], and dichloromethane and ethyl acetate leaf extracts against the chloroquine-sensitive strains 3D7 and D10, and the chloroquine-resistant strain W2, of *Plasmodium falciparum* [43,58,140]; the potent in vitro nematocidal activity of a methanol extract of the aerial parts and its partitions against the larvae of the root-knot nematode *Meloidogyne incognita*; the significant in vitro anti-COVID-19 activity of 95% ethanol extracts of the leaves and flowers from different cultivars [103]; the significant cancer reduction and increased survival rate of mice exhibited by a methanol leaf extract [141]; the high in vitro cytotoxicity of different leaf extracts [43,58,142]; the DNA-protective effects of an aqueous leaf extract [143]; the significant hepatoprotective effects of a methanol leaf extract [130]; the high insecticidal, larvicidal, and termiticidal activities of different extracts, especially of the leaves in polar solvents, against several insects (mosquitos, moths, termites, weevils, and bugs) [52,59,86,144,145,146,147,148,149,150,151,152,153]; the wound-healing effects of ethanol and water leaf extracts [108,154].

In summary, the alcoholic and aqueous leaf extracts seem to exhibit the highest and widest biological properties. In our opinion, the most promising biological effects of the extracts, which have attracted the greatest interest from several research groups, are the antiparasitic, nematocidal, and insecticidal properties.

Concerning the bioactivities of the compounds isolated from *L. camara* and the semisynthetic derivatives (Table 9), potent nematocidal effects against *Meloidogyne incognita* larvae (mortality ˃ 80%) were shown by different oleanane triterpenoids, such as camaric acid (**62**), camarin (**23**), camarinin (**56**), lantanilic acid (**68**), lantanolic acid (**27**), the ursane triterpenoids camarinic acid (**114**), lantacin (**119**), lantic acid (**102**), lantoic acid (**105**), pomolic acid (**107**), and ursolic acid (**106**), and the flavonoids linaroside (**139**) and lantanoside (**140**). Other interesting properties were the in vitro antiparasitic activity (IC_50_ < 10 μM) towards *Leishmania mexicana* promastigotes exhibited by camaric acid (**62**) and lantanilic acid (**68**). On the other hand, the in vitro cytotoxicity of most triterpenoids towards different human tumor cell lines was from moderate to very weak (IC_50_ = 20–80 μM) or null (IC_50_ ˃ 100 μM), except for the high activity (IC_50_ < 10 μM) exhibited by camaric acid (**62**), lantacamaric acid B (**70**), and lantanilic acid (**68**) towards HL-60 (JCRB0085) leukemia cells, icterogenin (**67**) towards colon cancer HCT-116 and lymphocytic leukemia L1210 cells, lantadene B (**61**) towards lung carcinoma A549 cells, oleanolic acid (**31**) towards drug-resistant human ovarian carcinoma IGROV-1 cells, and oleanonic acid (**22**) towards leukemia HL-60 and Ehrlich ascites carcinoma (EAC) cells. However, the in vivo antitumor activity, measured by the percent mice survival and percent overall papilloma incidence, was observed only for large doses of the administered compound, such as, for example, the ester **78**. Interesting in vivo antidiabetic effects were exhibited by urs-12-en-3*β*-ol-28-oic acid 3-*O*-β-D-glucopyranosyl-4′-octadecanoate (**127**). In vitro high antibacterial activity against the *Mycobacterium tuberculosis* strain H37Rv was exhibited by acetyl lantanoside (**144**). Powerful in vitro protein tyrosine phosphatase inhibition effects (IC_50_ < 10 μM) were determined for camaric acid (**62**), di(2-ethylhexyl) phthalate (**166**), 24-hydroxylantadene B (**65**), 22β-hydroxy-oleanolic acid (**32**), 22β-hydroxy-oleanonic acid (**25**), lantadenes A (**71**), B (**62**) and D (**54**), lantanilic acid (**68**), oleanolic acid (**31**), oleanonic acid (**22**), and reduced lantadenes A (**75**), B (**76**), and C (**77**). The in vitro anti-inflammatory activity was determined by measuring the inhibition of the following two inflammatory mechanisms: 22β-hydroxy-oleanonic acid (**26**) and lantadene A (**60**) and B (**61**) strongly inhibited the TNF-α-induced NF-ΚB activation (IC_50_ < 10 μM), but they were ineffective (IC_50_ ˃ 100 μM) against cyclooxygenase-2 (COX-2).

*L. camara* is also known for the toxicity to animals causing hepatotoxicity, photosensitization, and jaundice. Lantadene A (**60**) is the main toxic pentacyclic triterpenoid present in this weed.

**Table 8:** Biological activities of different extracts of *Lantana camara* L. ^a,^*.

Biological Activity	Part of the Plant/Solvent	Model	Results	Reference
Analgesic, anti-inflammatory	Leaves/water	The anti-inflammatory activity assay was carried out using carrageenan-induced lung edema and pleurisy mice. An analgesic effect assay was carried out using the formalin pain test.	Significant (*p* < 0.05) anti-inflammatory and analgesic activity, and minimal toxic effects.	[136]
Antibacterial	Leaves/ dichloromethane–methanol (1:1)	The in vitro antibacterial activity of a crude extract was screened at concentrations of 1000 μg/mL and 500 μg/mL against *Bacillus cereus* var *mycoides* (ATCC 11778), *B. pumilus* (ATCC 14884), *B. subtilis* (ATCC 6633), *Bordetella bronchiseptica* (ATCC 4617), *Micrococcus luteus* (ATCC 9341), *Staphylococcus aureus* (ATCC 29737), *S. epidermidis* (ATCC 12228), *Escherichia coli* (ATCC 10536), *Klebsiella pneumoniae* (ATCC 10031), *Pseudomonas aeruginosa* (ATCC 9027), and *Streptococcus faecalis* (MTCC 8043).	Except for *E. coli* and *P. aeruginosa*, a complete inhibition of bacterial growth was observed at both concentrations.	[60]
	Leaves, stems, and roots/methanol	The in vitro antibacterial activity of a crude extract was screened against *Bacillus cereus* (ATCC 14579), *Mycobacterium fortuitum* (ATCC 6841), and *Staphylococcus aureus* (ATCC 6538).	*B. cereus* and *M. fortuitum*: MIC and MBC values > 1000 μg/mL; *S. aureus*: MIC = 250 μg/mL, MBC > 1000 μg/mL.	[44]
	Leaves/methanol	A crude extract was tested in vitro with the disk diffusion method against *Escherichia coli*, *Proteus vulgaris*, and *Vibrio cholerae*.	Inhibition zone diameter = 50 mm.	[121]
	Leaves/ methanol, ethanol, water	Crude extracts were tested against *Bacillus subtilis* (ATCC 6059), *Klebsiella pneumoniae*, *Staphylococcus aureus* (ATCC 6538), and *Pseudomonas aeruginosa* (ATCC 7221).	MIC values (mg/mL) of a methanol extract: *B. subtilis* and *K. pneumoneae* = 8; *S. aureus* and *P. aeruginosa* = 5. MIC values (mg/mL) of an ethanol extract: *B. subtilis* = 10, *K. pneumoneae* = 12, *S. aureus* = 6.5, *P. aeruginosa* = 8. MIC values (mg/mL) of an aqueous extract: *B. subtilis* and *S. aureus* = 8, *P. aeruginosa* = 10.	[137]
	Flowers/70% aqueous ethanol	A crude extract was tested against the *Mycobacterium tuberculosis* H37RV strain at different concentrations (25, 50, and 100 μg/mL).	All concentrations inhibited the growth of *M. tuberculosis* H37RV from the first to the sixth week.	[138]
	Leaves/methanol	Crude extracts were tested against the *Mycobacterium smegmatis* mc2155 strain, *M. tuberculosis* H37Rv strain, and rifampicin-resistant *M. tuberculosis* TMC-331 strain.	MIC values (μg/mL) determined for *M. smegmatis* mc2155 strain = 574 ± 196; *M. tuberculosis* H37Rv strain = 574 ± 196; *M. tuberculosis* TMC-331 strain = 176 ± 33.	[155]
	Aerial parts/methanol	The in vitro antibacterial activity of a crude extract was tested against *E. coli* (ATCC25922), *Klebsiella pneumoniae*, *Pantoea sp*., and *Shigella flexneri.*	MIC values determined for all tested bacteria = 25 μg/mL.	[156]
	Leaves/methanol	A crude extract was tested with the disk diffusion method against *Helicobacter pylori*.	Inhibition zone diameter = 20 mm.	[128]
	Leaves and flowers/water, methanol, acetone, benzene	Crude flower and leaf extracts showed the highest inhibitory effects against *B. subtillis.* Extracts separated by column chromatography displayed weaker inhibitory effects against *B. subtillis* than crude extracts.	Inhibition area ranging from 6 to 9 mm. Inhibition area ranging from 3 to 7 mm.	[157]
Anticoagulant	Leaves, flowers, and roots/70% aqueous ethanol	The in vitro anticoagulant activity of crude extracts was tested at concentrations of 0.125, 0.25, 0.50, and 1 mg/mL.	The concentration of 1 mg/mL exhibited the highest anticoagulant activity; values expressed as prothrombin time: flowers = 21.7 ± 3 s; leaves = 16.9 ± 2.4 s; roots = 21.5 ± 2.8 s.	[158]
Antidiabetic	Leaves/water	Wistar albino rats (150–200 g); dose 1 = 250 mg extract/kg body weight and dose 2 = 500 mg extract/kg body weight were administered orally for 21 days.	Blood glucose levels: dose 1: at the 8th day = 183.83 ± 4.29 mg/dL; at the 14th day = 165.50 ± 4.26 mg/dL; at the 21st day = 136.83 ± 1.99 mg/dL. Dose 2: at the 8th day = 180.50 ± 3.07 mg/dL; at the 14th day = 157.83 ± 5.28 mg/dL; at the 21st day = 124.67 ± 2.40 mg/dL.	[125]
Antidiarrheal	Leaves/ethanol	Group II of male mice (Laca strain; 20–25 g) received 1% leaf powder for ten days; groups III–VI received a single dose of 125, 250, 500, and 1000 mg extract/kg body weight; subsequently, castor oil-induced diarrhea was evaluated.	% Intestinal transit: group II: 34.78 ± 3.52; group III: 1 ± 0.01; group IV: 26.46 ± 6.82; group V: 31.74 ± 1.49; group VI: 38.67 ± 6.60. Mean defecation in 4 h: group III: 9 ± 1.18; group IV: 9 ± 2.06; group V: 1 ± 0.05; group VI: total constipation.	[100]
	Leaves/80% aqueous methanol	Groups III–V of Swiss albino mice (6–88 weeks; 20–30 g) received a single dose of 100, 200, and 400 mg extract/kg body weight; subsequently, castor oil-induced diarrhea was evaluated.	The most effective dose was 400 mg/kg body weight with a 67.9% inhibition of diarrhea and an antidiarrheal index of 87.6.	[159]
Antifungal	Leaves/dicloromethane–methanol (1:1)	The antifungal activity of a crude extract was tested at concentrations of 1000 μg/mL and 500 μg/mL against *Candida albicans* (MTCC 10231), *Aspergillus niger* (MTCC 1344), and *Saccharomyces cerevisiae* (ATCC 9763).	Complete inhibition was observed at both concentrations.	[60]
	Leaves, stems, and roots/methanol	The in vitro antifungal activity of a crude extract was tested against *Candida albicans* (ATCC 10231).	MIC and MBC values > 1000 μg/mL.	[44]
	Leaves/methanol	A crude extract was tested in vitro with the disk diffusion method against *Aspergillus niger* and *Candida albicans*.	Inhibition zone diameter = 0.5 mm.	[79]
	Leaves/methanol, water	Crude extracts were tested against *Aspergillus fumigatus* and *A. flavus*.	% Inhibition of the methanol extract: *A. fumigatus* = 71.4; *A. flavus* = 66.4. % Inhibition of the water extract: *A. fumigatus* = 61.5; *A. flavus* = 57.8.	[137]
Antioxidant	Leaves, flowers, fruits, roots, and stems/methanol	Phytochemical analysis: Folin–Ciocalteu assay; gallic acid and ascorbic acid as reference standard. The in vitro antioxidant activity was tested by the following different methods: 1,1-diphenyl-2-picrylhydrazyl (DPPH) radical scavenging assay, with ascorbic acid as a reference standard; xanthine oxidase inhibition assay, with allopurinol as a reference standard; Griess–Ilosvay method, with allopurinol as a reference standard.	Leaves: total phenols > 100 mg/g extract; DPPH assay: IC_50_ = 16.02 ± 0.04 μg/mL; xanthine oxidase inhibition assay: IC_50_ < 20 μg/mL; Griess–Ilosvay method: IC_50_ < 10 ± 2 μg/mL. Flowers: total phenols > 100 mg/g extract; DPPH assay: IC_50_ = 28.92 ± 0.19 μg/mL; xanthine oxidase inhibition assay: IC_50_ > 20 μg/mL; Griess–Ilosvay method: IC_50_ < 10 ± 2 μg/mL. Fruits: total phenols < 100 mg/g extract; DPPH assay: IC_50_ = 90.11 ± 0.57 μg/mL; xanthine oxidase inhibition assay: IC_50_ > 20 μg/mL; Griess–Ilosvay method: IC_50_ > 10 ± 2 μg/mL. Roots: total phenols > 100 mg/g extract; DPPH assay: IC_50_ = 31.52 ± 0.74 μg/mL; xanthine oxidase inhibition assay: IC_50_ > 20 μg/mL; Griess–Ilosvay method: IC_50_ < 10 ± 2 μg/mL. Stems: total phenols > 100 mg/g extract; DPPH assay: IC_50_ = 46.96 ± 2.51 μg/mL; xanthine oxidase inhibition assay: IC_50_ < 20 μg/mL; Griess–Ilosvay method: IC_50_ > 10 ± 2 μg/mL.	[139]
	Leaves and roots/ethanol	Phytochemical analyses: Folin–Ciocalteu assay, with gallic acid as a reference standard; aluminum chloride method, with quercetin as a reference standard; quantification of phenolics and flavonoids by HPLC-DAD. In vitro antioxidant activity was determined by the following different methods: thiobarbituric acid reactive substances (TBARS) assay with phospholipids; iron chelation assay; deoxyribose degradation assay; ferric-reducing antioxidant power (FRAP).	Leaves: total phenols: 227.10 ± 9.07; Gallic Acid Equivalents (GAE μg/mg), 22.7%; total flavonoids: 46.55 ± 1.50; quercetin equivalents (QuercE μg/mg), 4.6%; caffeic acid (10.75 ± 0.04 mg/g), 1.07%; quercetin (2.87 ± 0.01 mg/g, 0.28%); TBARS assay: basal IC_50_ = 57.69 ± 4.01 μg/mL; induced Fe^2+^ IC_50_ = 32.48 ± 3.51 μg/mL; iron chelation assay: IC_50_ = 214.20 ± 2.50 μg/mL; deoxyribose degradation assay: IC_50_ = 285.64 ± 20.63 μg/mL; FRAP assay: 8.28 ± 0.07 mM Fe ^2+^/g extract. Roots: total phenols = 211.80 ± 7.94 (GAE μg/mg), 21.3%; total flavonoids = 33.64 ± 1.52 (QuercE μg/mg, 3.3%); caffeic acid (8.27 ± 0.01 mg/g, 0.82%), rutin (5.35 ± 0.03 mg/g, 0.53%); TBARS assay: basal IC_50_ = 168.92 ± 7.36 μg/mL; induced Fe^2+^ IC_50_ = 63.84 ± 4.56 μg/mL; iron chelation assay: IC_50_ = 448.19 ± 4.50 μg/mL; deoxyribose degradation assay: IC_50_ = 276.89 ± 31.26 μg/mL; FRAP assay: 11.64 ± 0.10 mM Fe^2+^/g extract.	[160]
	Leaves/methanol	The concentration of lipid peroxides (LPOs) in the stomach mucosa of Wister albino rats used in ulcerogenic models was indirectly measured by the TBARS assay; the concentration of reduced glutathione was also determined.	LPO: 29.23 ± 0.35 and 27.7 ± 0.50 nmol/g; GSH: 181.52 ± 0.83 and 202.9 ± 1.08 μg/g.	[128]
	Leaves/methanol	DPPH assay.	IC_50_ = 74.3 μg/mL.	[130]
	Leaves/methanol	Quantitative analysis of phytochemicals: total phenolic content, total flavonoid content, and total tannin content. In vitro antioxidant activity (DPPH radical scavenging activity assay and hydroxyl radical scavenging activity assay).	Total phenolic content: 40.859 ± 0.017 (mg GAE/g dry sample). Total flavonoid content: 53.112 ± 0.199 (mg rutin/g dry sample). Total tannin content: 0.860 ± 0.038 (mg/g dry sample). DPPH assay: IC_50_ ≥ 0.2 mg/mL. Hydroxyl radical assay: IC_50_ ≤ 0.2 mg/mL.	[137]
	Leaves/ethyl acetate	Total phenolic content and DPPH assay.	Total phenolic content: 2419.6 mg/L GAE. DPPH assay: IC_50_ = 36.18 mg/mL.	[161]
	Aerial parts/methanol	The antioxidant capacity of a crude extract was evaluated by the FRAP and the DPPH assays.	FRAP: 8.17 ± 0.04 mmol/g, DPPH: IC_50_ = 16.13 ± 0.35 μg/mL.	[156]
	Leaves/water	DPPH, metal chelating activity, and FRAP assays.	DPPH assay: IC_50_ = 42.66 μg/mL; metal chelating activity assay: IC_50_ = 1036.4 μg/mL; FRAP test: dose-dependent activity.	[162]
	Leaves/methanol	DPPH and FRAP assays.	DPPH: IC_50_ = 24.80 ± 0.52 μg/mL; FRAP: IC_50_ = 21.61 ± 0.26 μg/mL.	[143]
Antiparasitic	Leaves and stems/dichloromethane; dichloromethane–methanol (1:1); water	*Leishmania amazonensis* (MHOM/77BR/LTB0016) promastigotes and amastigotes.	Dichlorometane extract: IC_50_ = 11.7 ± 4.4 μg/mL and IC_50_: 21.8 ± 2.4 μg/mL; dichloromethane–methanol (1:1) and water extracts: IC_50_ > 200 μg/mL.	[64]
	Leaves/methanol Aerial parts/dichloromethane	*Leishmania amazonensis* (MHOM/Br/75/Josefa) isolated promastigotes and *L. chagasi* (MHOM/Br/74/PP75) isolated promastigotes. The in vitro antiparasitic activity of a crude extract and 18 fractions obtained by open-column chromatographic separation were tested against *Leishmania amazonensis* (MHOM/BR/77/LTB0016) promastigotes and *L. mexicana* isolated amastigotes.	IC_50_ = 14 μg/mL and IC_50_ > 250 μg/mL. Crude extract: IC_50_ = 21.8 ± 2.4 μM and IC_50_ = 42.6 ± 1.9 μM. The most active fractions against *L. amazonensis* amastigotes were as follows: FII, IC_50_ = 9.1 ± 3.4 μM; FX, IC_50_ = 7.9 ± 0.3 μM; FXI, IC_50_ = 8.0 ± 1.1 μM; FXVI, IC_50_ = 8.5 ± 1.7 μM.	[67] [109]
	Leaves/dichloromethane	Dichloromethane extracts of two different batches were tested against the chloroquine-sensitive strain 3D7 and chloroquine-resistant strain W2 of *Plasmodium falciparum*, and by the parasite dehydrogenase lactate essay. Five female Swiss mice (10 weeks old; 25 ± 2 g), infected by *Plasmodium berghei* NK173, received a single dose of 50 mg extract/kg body weight daily for 4 days intraperitoneally.	3D7: IC_50_ = 8.7 ± 1 and 14.1 ± 8.4 μg/mL; W2: IC_50_ = 5.7 ± 1.6 and 12.2 ± 2.9 μg/mL. 5% growth inhibition.	[43]
	Leaves/ethyl acetate	The extract was tested on chloroquine-resistant strains (3D7 and INDO) of *Plasmodium falciparum.*	3D7: IC_50_ = 19 ± 0.57 μg/mL; INDO: IC_50_ = 20 ± 1.5 μg/mL.	[58]
	Leaves and twigs/dichloromethane–methanol (1:1); water	Crude extracts were tested in vitro against the chloroquine-sensitive strain D10 of *Plasmodium falciparum* and by the parasite dehydrogenase lactate assay.	IC_50_ = 11 μg/mL; IC_50_ < 1000 μg/mL.	[140]
	Aerial parts/methanol	The in vitro nematocidal activity of crude extract and its partitions were screened against *Meloidogyne incognita* larvae, at concentrations of 0.5%, 0.25%, and 0.125% after 48 h.	At a 0.5% concentration, the methanol extract: 85% mortality; ether insoluble partition: 90% mortality; methanolic acidic partition: 88% mortality; ether soluble partition: 75% mortality; *n*-hexane soluble partition: 60% mortality; *n*-hexane insoluble partition: 50% mortality.	[99]
Antiulcerogenic	Leaves/methanol	Wister albino rats (150–200 g) were divided into 4 groups; groups 2 and 3 received 250 and 500 mg extract/kg orally. Aspirin-induced ulcerogenesis in a pyloric ligated system (APL); ethanol-induced ulcer model (EIM); cysteamine-induced duodenal ulcer model (Cys).	Ulcer index inhibition: APL: 46.61% and 73.97%; EIM: 55.60% and 63.39%; CYS: 41.43% and 68.90%.	[103]
Antiviral	Leaves and flowers/95% ethanol	The in vitro anti-COVID-19 activity of crude extracts from different cultivars were screened by the plaque reduction assay.	Spreading sunset cultivar: flowers: IC_50_ = 4.188 μg/mL; leaves: IC_50_ = 8.751 μg/mL. Chelsea gem cultivar: flowers: IC_50_ = 3.671 μg/mL; leaves: IC_50_ = 3.181 μg/mL. Nivea cultivar: flowers: IC_50_ = 15.050 μg/mL; leaves: IC_50_ = 6.820 μg/mL. Drap d’or cultivar: flowers: IC_50_ = 5.015 μg/mL; leaves: IC_50_ = 8.715 μg/mL.	[103]
Anxiolytic	Leaves/methanol	Ursolic acid stearyl glucoside (UASG) was isolated from the leaves of *L. camara* using column chromatography. The compound was administered to the animals in a dose-dependent manner to evaluate its effects at different concentrations	A dose-dependent effect, with higher doses of UASG (25 and 50 mg/kg) leading to more pronounced anxiolytic effects. UASG reduced the anxiety and increased the locomotor activity. The anxiolytic effects of UASG were comparable to those of diazepam (1 mg/kg), a standard anxiolytic drug, indicating that UASG may have a similar therapeutic potential.	[163]
Chemoprotective effect	Leaves/methanol	Female Swiss albino mice (6 weeks old; 18–22 g). Group III received 400 mg extract/kg body weight, which was given orally as a suspension in water and carboxymethyl cellulose, twice a week (100 nmol/100 μL), applied for 20 weeks topically.	A significant reduction in cancer (76.4%) was observed, and the survival rate was 75%.	[141]
Cytotoxic	Leaves and stems/dichloromethane (a); dichloromethane–methanol (1:1) (b); water (c).	BALB/c mice peritoneal macrophages.	(a): CC_50_ > 100 μg/mL; (b): CC_50_ > 200 μg/mL; (c): CC_50_ = 125.9 ± 3.1 μg/mL.	[64]
	Leaves/dichloromethane	The cytotoxicity of dichloromethane extracts from two different batches was tested in vitro towards normal human fetal lung fibroblasts WI-38.	IC_50_ = 69.5 ± 12.1 μg/mL and IC_50_ = 97.2 ± 2.4 μg/mL.	[43]
	Leaves/ethyl acetate	HeLa cells and the MTT assay.	IC_50_ = 42 ± 2.3 μg/mL.	[58]
	Leaves/ethanol	Tested towards Hela cancer cells.	IC_50_ = 43.54 μg/mL.	[142]
	Leaves/methanol	Vero cells.	IC_50_ values at 24 h exposure = 361.44 ± 10.68 μg/mL; at 48 h exposure = 319.37 ± 99.80 μg/mL.	[103]
DNA protection	Leaves/water	H_2_O_2_ photolysis by UV radiation in the presence of pBR322 plasmid DNA and an aqueous extract (50 g).	Treatment with the extract at the evaluated dose completely protected the plasmid DNA.	[162]
Hemolytic	Leaves/water	The hemolytic activity of a crude extract and the hexane–ethyl acetate (50:50), chloroform, methanolic, and ethanolic partitions were screened at different concentrations (125, 250, 500, and 1000 μg/mL).	CC_50_ values (μg/mL): aqueous extract = 8035.9; hexane–ethyl acetate (50:50) phase = 4470.4; chloroform phase = 2739.8; methanolic phase = 12332.0; ethanolic phase = 9496.4.	[107]
Hepatoprotective effect	Leaves/methanol	In vivo acetaminophen-induced hepatotoxicity on a mice model. The mice of groups III and IV received a dose of 25 and 75 mg extract/kg daily for 7 days before receiving a single dose of acetaminophen. The mice of groups V and VI received a dose of 25 and 75 mg extract/kg daily for 7 days before receiving a single dose of acetaminophen. Subsequently, the serum glutamate oxaloacetate transaminase (SGOT), serum glutamate pyruvate transaminase (SGPT), and alkaline phosphatase (ALP) activities were measured.	Among all of the tested groups, pretreatment with a 75 mg extract/kg significantly reduced the SGOT = 144.5 ± 3.74 (UI/L), SGPT = 112.4 ± 9.1 (UI/L), and ALP = 96.8 ± 3.2 (UI/L) activities compared to control groups.	[130]
	Leaves/methanol (Lantadenes concentrated fraction)	A *Ginkgo biloba* methanolic extract (GBME) was evaluated against lantadenes-induced hepatic damage in guinea pigs. Guinea pigs (200–250 g) were divided into 5 groups. Group I: negative control; group II received 25 mg lantadenes/kg body weight; group III received 25 mg lantadenes/kg body weight + 100 mg GBME/kg body weight; group IV received 25 mg lantadenes/kg body weight + 200 mg GBME/kg body weight; group V: positive control, received 100 mg silymarin/kg body weight. The drugs were administered orally in gelatin capsules daily for 14 days. Analysis by HPLC of the lantadenes fraction (72.82% lantadene A).	Serum protein levels of group IV were significantly lower than group II.	[164]
Insecticidal/larvicidal/termiticidal	Leaves/methanol, *n*-hexane	Methanol and *n*-hexane extracts were tested in vivo against *Anopheles stephensi* (Liston).	The methanol extract was more active than the *n*-hexane extract. The optimal dose for the repellent activity was 2 mg/mL.	[52]
	Aerial parts/ethanol	*Phthorimae operculella* (Zeller).	High insecticidal effect against *Phthorimae operculella* (Zeller); no ovocidal effects.	[144]
	Leaves/ethyl acetate, methanol	Tested against *Anopheles stephensi* (Liston) and *Culex quinquefasciatus* (Say) larvae.	Ethyl acetate extract: 500 ppm, 30 min: 98% mortality and 500 ppm, 30 min: 93% mortality. Methanol extract: 500 ppm, 30 min: 82% mortality and 500 ppm, 30 min: 86% mortality.	[145]
	Whole plant/ethanol	The in vitro larvicidal activity of the methanol and petroleum ether partitions from extracts of different parts of the plant were assayed with the brine shrimp lethality test.	Methanol partitions: leaves: LC_50_ = 18 μg/mL; roots: LC_50_ = 17 μg/mL; twigs and stems: LC_50_ = 0.3 μg/mL. Petroleum ether partitions: leaves: LC_50_ = 54 μg/mL; roots: LC_50_ = 47 μg/mL; twigs: LC_50_ = 62 μg/mL; stems: LC_50_ = 3.6 μg/mL.	[86]
	Leaves/chloroform	The termiticidal activity of several extracts was screened against *Microcerotermes beesoni.*	Most active extract: LD_50_ = 5 μg/insect.	[146]
	Leaves and seeds/powder	Applied to *Zea mays* L. against *Sitophilus zeamais.*	63.3% mortality of *Sitophilus zeamais* on the twenty-eighth day.	[147]
	Leaves/*n*-hexane	Different concentrations (10%, 5%, 2.5%, 1.25%, 0.1%, 0.05%, 0.025%, 0.0125%, and 0.00625%) of a crude extract were tested against *Dysdercus koenigii* Fabricius nymphs for 24 h and monitored for 7 days.	Survival of nymphs at 10%, 5%, 2.5%, and 1.25% concentrations = 65.33%, 66.67%, 72%, and 85.33%. Reduction in the longevity at 10% and 5% concentrations = 5.54 and 5.95 days.	[148]
	Leaves and flowers/ethanol	Crude extracts were tested against *Anopheles arabiensis* and *Culex quinquefasciatus* larvae.	Flowers: *A. arabiensis*, LC_50_ = 15.84 ppm; *C. quinquefasciatus*, LC_50_ = 21.37 ppm. Leaves: *A. arabiensis*, LC_50_ = 9.54 ppm; *C. quinquefasciatus*, LC_50_ = 5.01 ppm.	[149]
	Whole plant/water, acetone, chloroform, ethanol, and methanol	The larvicidal activity of different concentrations (25, 50, 75, 100, and 150 ppm) of crude extract was screened for 24 h against *Aedes aegypti*, *Anopheles stephensis*, and *Culex quinquefasciatus*.	The most active extracts: methanol: *A. aegypti*, LC_50_ = 39.54 ppm; *A. stephensis*, LC_50_ = 35.65 ppm; *C. quinquefasciatus*, LC_50_ = 35.36 ppm; ethanol: *A. aegypti* LC_50_ = 60.93 ppm; *A. stephensi*, LC_50_ = 79.03 ppm; *C. quinquefasciatus*, LC_50_ = 50.17 ppm.	[150]
	Leaves/diluted aqueous juice	Different concentrations (25, 50, 75, and 100 ppm) were tested against *Aedes aegypti*, *Anopheles subpictus*, and *Culex quinquefasciatus* larvae during 6, 12, and 24 h.	LC_50_ values ranged from 47.47 to 101.68 ppm.	[151]
	Leaves/acetone	The insecticidal activity of different concentrations (100, 200, 300, 400, and 500 ppm) of a crude extract was tested against *Aedes aegypti* L. larvae and pupae for 24 h.	Larvae: LC_50_ = 198.52 ppm; pupae: LC_50_ = 309.64 ppm.	[59]
	Leaves/water	The insecticidal activity of different concentrations (62.5, 125, 250, 500, and 1000 ppm) of a crude extract was tested against *Aedes aegypti* L. and *Culex quinquefasciatus* Say larvae for 24 h.	*A. aegypti* L.: LC_50_ = 35.48 ppm; *C. quinquefasciatus*: LC_50_ = 35.19 ppm.	[152]
	Leaves/95% ethanol	Different concentrations (250–3000 ppm) of a crude extract were tested against *Anopheles arabiensis* Patton larvae.	LC_50_ = 477.53 ppm.	[153]
Phytotoxic	Leaves/water	Different concentrations (0%, 1.25%, 2.5%, 3.75%, and 5%, *v*/*v*) of a crude extract were tested on *Bidens pilosa* seeds.	The aqueous extract reduced the viability of *Bidens pilosa* seeds during phase III of germination. At any concentration, the aqueous extract inhibited the root and epicotyl growth.	[154]
	Leaves/methanol–water (70:30)	A crude extract, at the concentration of 5 g/L, was tested on *Eichhornia crassipes* (Mart.) Solms and *Microcystis aeruginosa* Kütz.	*E. crassipes*: complete inhibition; *M. aeruginosa*: 66.1% inhibition.	[110]
	Leaves and callus/water	The inhibition of seed germination by crude extracts was tested on *Brassica campestris* var. *chinensis*, *Ipomoea aquatica* Forsk., *Sorghum bicolor* L., and *Zea mays* L.	Extract concentration that caused 50% inhibition of seed germination: *B. campestris*: leaves = 0.62%, callus = 0.65%; *I. aquatica*: leaves = 0.94%, callus = 0.45%; *S. bicolor*: leaves = 0.95%, callus = 1.19%; *Z. mays*: leaves = 4.39%, callus = 3.05%.	[165]
	Leaves/water	An aqueous leaf leachate was tested on *Eichhornia crassipes* (Mart.) Solms.	The concentration of 5% was the most toxic after 21 days.	[166]
	Callus/water	An aqueous leaf leachate was tested on *Salvinia molesta* Mitchell.	The concentration of 1% was the most toxic after 7 days.	[167]
	Callus/water	A crude extract encapsulated in calcium alginate beads was tested on *Brassica campestris* var. *chinensis.*	Beads with 5% extract had no effect on the germination rate, while beads with 1–4% extract did not reduce the total weight of fresh seedlings.	[168]
Wound-healing effects	Leaves/water Leaves/ethanol	Daily topical application of 100 mg extract/kg body weight on wounds of male Sprague Dawley rats (200–220 g). Bovine dermatophilosis caused by *Dermatophilus congolensis* was treated with ointments containing *L. camara* leaf ethanolic extracts once a day for 10 days.	Mean epithelization time and % of wound healing: placebo group = 19 ± 0.14 days and 88%; tested group = 17.20 ± 0.12 days and 98%. Wound healing was observed between the third and fourth day of application without recurrence.	[169] [108]

^a^ Biological activities are ordered in alphabetic order. * MIC = minimum inhibitory concentration; MBC = minimum bactericidal concentration; IC_50_ = sample concentration causing 50% inhibition; LC_50_ = sample concentration that causes 50% mortality; CC_50_ = sample concentration causing 50% cytotoxicity.

**Table 9:** Bioactivities determined for the compounds isolated from *Lantana camara* and semisynthetic derivatives.

Compound ^a,^* (Nº)	Biological Activity	Reference
Acetyl lantanoside * (**144**)	In vitro antibacterial activity against *Mycobacterium tuberculosis* strain H37Rv (ATCC 27294): 98% inhibition, MIC < 11.15 μM.	[16,134]
22*β*-Acetyloxy-oleanonic acid * (**37**)	In vitro cytotoxic activity towards human leukemia HL-60 cells: IC_50_ = 75.09 ± 0.09 μM; human cervical carcinoma Hela cells: IC_50_ = 72.75 ± 0.29 μM; colon 502,713 cells: IC_50_ = 67.1 ± 0.04 μM; lung carcinoma A549 cells: IC_50_ = 71.77 ± 0.15 μM.	[94]
22*β*-Benzoyloxy-oleanonic acid * (**85**)	In vitro cytotoxic activity towards HL-60 cells: IC_50_ = 88.38 ± 0.15 μM; Hela cells: IC_50_ = 80.55 ± 0.15 μM; colon 502,713 cells: IC_50_ = 89.07 ± 0.04 μM; lung A549 cells: IC_50_ > 100 μM.	[94]
22*β*-Butanoyloxy-oleanonic acid * (**50**)	In vitro cytotoxic activity towards HL-60 cells: IC_50_ = 39.94 ± 0.23 μM; Hela cells: IC_50_ = 42.16 ± 0.15 μM; colon 502,713 cells: IC_50_ = 46.6 ± 0.28 μM; lung A549 cells: IC_50_ = 50.11 ± 0.09 μM. In vivo antitumor activity: squamous cell carcinogenesis induced by 7,12-dimethylbenz[a]anthracene/12-*O*-tetradecanoylphorbol-13-O-acetate in Swiss albino mice (LACCA/female); 50 mg/kg body weight administered orally for 20 weeks: 80% mice survival and 17.2% overall papilloma incidence.	[94]
**Camaric acid** (**62**)	In vitro nematocidal activity towards *Meloidogyne incognita* larvae: 95% mortality at 0.5% concentration after 48 h. In vitro antiparasitic activity towards *Leishmania mexicana* promastigotes: IC_50_ = 2.52 ± 0.08 μM. In vitro protein tyrosine phosphatase 1B inhibition assay: IC_50_ = 5.1 μM [84]. In vitro cytotoxic activity towards HL-60 human promyelocytic leukemia cells (JCRB0085): IC_50_ = 1.71 ± 0.10 μM. In vitro anti-inflammatory activity (inhibition of LPS-induced NO production in BV-2 cells): IC_50_ > 3 μM.	[80,82,84,99,117,118]
**Camarin** (**23**)	In vitro nematocidal activity against *Meloidogyne incognita* larvae: 100% mortality at 1 mg/mL concentration after 48 h.	[90]
**Camarinic acid** (**114**)	In vitro antimicrobial and antifungal activity index values: *E. coli* = 2, *S. aureus* = 0.95, *P. aeruginosa* = 0.15, *S. typhi* = 0.7, *C. albicans* = 0.2, *T. mentagrophytes* = 2.3. In vivo antimutagenic evaluation: micronucleus test (2.75 mg mitomycin D/kg body weight and 6.75 mg/kg body weight given orally to Swiss strain mice, once a day-48 h): 76.7% reduction in the number of micronucleated polychromatic erythrocytes. In vitro nematocidal activity against *Meloidogyne incognita* larvae: 100% mortality at 1% concentration after 24 h. In vitro antiparasitic activity against *Leishmania major* promastigotes: IC_50_ = 89 ± 0.3 μM.	[16,78]
**Camarinin** (**56**)	In vitro nematocidal activity against *Meloidogyne incognita* larvae: 100% mortality at 1 mg/mL concentration after 48 h.	[90]
**Di-(2-ethylhexyl) phthalate** (**166**)	In vitro antibacterial activity (disk diffusion method), zone inhibition diameter: *Escherichia coli* = 20 mm, *Staphylococcus aureus* = 22 mm, *Salmonella typhimurium* = 21 mm, *Pseudomonas aerugionosa =* 23 mm. In vitro protein tyrosine phosphatase inhibition assay: IC_50_ = 8.1 μM.	[73,84]
**Ethyl-*β*-D-galactopyranoside** (**163**)	Inactive in an in vitro antiparasitic activity assay towards *Brugia malayi.*	[74]
**3-*O-*β-D**-**Glucosyl oleanolic acid** (**84**)	In vivo antiulcer activity: aspirin-induced and ethanol-induced ulcer models; Albino Wistar rats (150–200 g) were divided into 4 groups. Groups III and IV received 25 and 50 mg compound/kg body weight, respectively, orally once a day for 5 days. Ulcer index: 3.48± 0.83 and 1.99 ± 0.34, respectively; protection: 21.24 and 38.37%, respectively.	[126]
**Hispidulin** (**128**)	In vitro protein tyrosine phosphatase inhibition assay: IC_50_ > 33 μM.	[72,79,84]
**9-Hydroxy-lantadene A** (**64**)	In vitro antifungal activity against *Fusarium subglutinans* (PPRI 6740), *F. solani* (PPRI 19147), *F. graminearum* (PPRI 10728), and *F*. *semitectum* (PPRI 6739): MIC ˃ 1000 μM; against *F. proliferatum* (PPRI 18679): MIC = 70.32 μM. In vitro cytotoxic activity towards Raw 264.7 cells: IC_50_ ˃ 100 μM.	[120]
**24-Hydroxy-lantadene B** **≡ 24-Hydroxy-22*β*-senecioyloxy-oleanonic acid** (**65**)	Binding affinity to the antiapoptotic protein Bcl-xL: Ki = 5.3 μM. In vitro cytotoxic activity towards papilloma KB cells: IC_50_ = 35.5 μM; colon carcinoma HCT-116 cells: IC_50_ = 11.4 μM; breast adenocarcinoma MCF7 cells: IC_50_ = 42.5 μM; lymphocytic leukemia L1210 cells: IC_50_ = 12.3 μM. In vitro protein tyrosine phosphatase inhibition assay: IC_50_ = 7.3 μM.	[84,116]
**24-Hydroxy-lantadene D** (**53**)	In vitro protein tyrosine phosphatase inhibition assay: IC_50_ >18 μM.	[84]
**22*β*-Hydroxy- oleanolic acid** (**32**)	In vitro cytotoxic activity: tested on multiple cancer cells. In vitro anti-inflammatory activity (TNF-α-induced NF-ΚB activation inhibitory activity): IC_50_ ˃10 μM; COX-2 inhibition: IC_50_ ˃100 μM. In vitro cytotoxic activity towards A549 cells: IC_50_ ˃10 μM. In vitro protein tyrosine phosphatase inhibition assay: IC_50_ = 7.9 μM.	[80,84,95,96]
**22*β*-Hydroxy-oleanonic acid** (**26**)	In vitro antitumor activity: Epstein–Barr virus early antigen activation assay induced by 12-*O*-tetradecanoylphorbol-13-*O*-acetate (TPA) in Raji cells: 35.3% inhibition at 100 mol tested compound/1 mol TPA. In vivo hepatotoxicity evaluation (adult female guinea pigs received 125 mg compound/kg body weight orally in gelatin capsules): bilirubin: 0.67 ± 0.001 mg/100 mL, SGOT: 46.1 ± 0.4 U/L, SGPT: 39 ± 0.3 U/L; nontoxic. In vitro cytotoxic activity towards HL-60, Hela, colon 502,713, and lung A549 cells: IC_50_ > 100 μM; A549 cells: IC_50_ ˃ 10 μM [94]. In vitro anti-inflammatory activity (inhibitory activity of TNF-α-induced NF-ΚB activation): IC_50_ = 6.42 ± 1.24 μM; COX-2 inhibition: IC_50_ ˃ 100 μM. In vitro protein tyrosine phosphatase inhibition assay: IC_50_ = 6.9 μM.	[84,92,93,94,95,96]
**11*α*-Hydroxy-3-oxo-urs-12-en-28-oic acid** (**101**)	In vitro nematocidal activity against *Meloidogyne incognita* larvae: 70% mortality at 0.25% concentration after 72 h.	[82]
**Icterogenin** (**67**)	Binding affinity to the antiapoptotic protein Bcl-xL: Ki *=* 7.6 μM. In vitro cytotoxic activity towards KB cells: IC_50_ = 15 μM; HCT-116 colon cancer cells: IC_50_ = 5.8 μM; MCF7 cells: IC_50_ = 11.3; L1210 lymphocytic leukemia cells: IC_50_ = 6.8 μM; HL-60 human promyelocytic leukemia cells (JCRB0085): IC_50_ = 34.2 ± 0.7 μM. In vitro protein tyrosine phosphatase inhibition assay: IC_50_ = 11 μM. In vitro anti-inflammatory activity (inhibition of LPS-induced NO production in BV-2 cells): IC_50_ > 3 μM. DPPH radical scavenging activity: IC_50_ = 169.7 μg/mL.	[109]
**Lancamarinic acid** (**41**)	In vitro screening against a variety of Gram-positive and Gram-negative bacteria (disk diffusion method).	[105]
**Lancamarolide** (**42**)	In vitro nematocidal activity against *Meloidogyne incognita* larvae: 80% mortality at 0.25% concentration after 48 h.	[81]
**Lantacamaric acid A** (**29**)	In vitro cytotoxic activity towards HL-60 human promyelocytic leukemia cells (JCRB0085): IC_50_ = 30.8 ± 2.7 μM.	[99]
**Lantacamaric acid B** (**70**)	In vitro cytotoxic activity towards HL-60 human promyelocytic leukemia cells (JCRB0085): IC_50_ = 6.60 ± 0.46 μM.	[99]
**Lantacin** (**119**)	In vitro nematocidal activity against *Meloidogyne incognita* larvae: 100% mortality at 1 mg/mL concentration after 48 h.	[81,90]
**Lantadene A (Rehmannic acid)** (**60**)	In vitro larvicidal activity: very toxic in the brine shrimp lethality test; insecticidal activity at 5.0 mg/mL towards *Spodoptera littoralis* Biosduval: 40% lethality after 48 h; fecundity inhibition assay in *Clavigralla tomentosicollis* Stal.: 50% fecundity suppression; inactive towards *Aphis craccivora* Koch. In vivo antimotility effect evaluation (Laca strain male mice (20–25 g) received a single injection of 85 and 170 mg compound/kg body weight): % intestinal transit = 39.47 ± 10.05 and 27.34 ± 4.58, respectively. Phytotoxic activity towards *Eichhornia crassipes* (Mart.) Solms and *Microcystis aeruginosa* Kutz: ErC_50_ = 24.78 and 21.34 mg/L, respectively. In vivo hepatotoxicity evaluation (adult female guinea pigs received 125 mg compound/kg body weight orally in gelatin capsules): bilirubin = 8.74 ± 2.5 mg/100 mL, SGOT = 696.3 ± 3.1 U/L, SGPT = 305.2 ± 3.9 U/L; toxic. In vitro cytotoxicity towards HL-60 cells: IC_50_ = 35.81 ± 0.40 and 35 ± 1 μM; HeLa cells: IC_50_ = 42.15 ± 0.09 and 42 ± 8 μM; colon 502,713 cells: IC_50_ = 38.53 ± 0.09 and 38 ± 5 μM μM; lung A549 cells: IC_50_ = 39.43 ± 0.21, 39 ± 1 μM μM, and 2.84 ± 0.72 μM; KB cells: IC_50_ = 15.8 μM; HCT-116 cells: IC_50_ = 41.8 μM; MCF7 cells: IC_50_ = 44.7 and ˃ 100 μM; L1210 cells: IC_50_ = 16.3 μM; HL-60 human promyelocytic leukemia cells (JCRB0085): IC_50_ = 25.4 ± 3.1 μM; LNcap prostatic cancer cells: IC_50_ ˃ 100 μM; RWPE-1 prostatic cancer cells: IC_50_ ˃ 100 μM. Lantadene A-gold nanoparticles reduced MCF-7 (breast cancer cells) viability, upregulated the p53 expression, and downregulated the BCL-2 expression. In vivo antitumor activity: squamous cell carcinogenesis induced by 7,12-dimethylbenz[a]anthracene-12-*O*-tetradecanoylphorbol-13-*O*-acetate in Swiss albino mice (LACCA/female); 50 mg compound/kg body weight administered orally for 20 weeks: 80–100% mice survival and 17.9–18.1% overall papilloma incidence. Binding affinity to the antiapoptotic protein Bcl-xL: Ki > 100 μM. Antioxidant activity in a dose-dependent manner. Toxicity evaluation: toxic (2 g) orally to sheep; nontoxic to lambs (167 mg compound/kg body weight administered orally in gelatin capsules) and guinea pigs (667 mg compound/kg body weight administered orally in gelatin capsules). In vitro antiparasitic activity against *Leishmania major* promastigotes: IC_50_ = 20.4 ± 0.1 μM. In vitro anti-inflammatory activity (inhibition of TNF-α-induced NF-ΚB activation): IC_50_ = 1.06 ± 0.46 μM; COX-2 inhibition: IC_50_ ˃ 100 μM. In vitro nematocidal activity towards *Meloidogyne incognita* larvae: 70% mortality at 0.5% concentration after 48 h. In vitro protein tyrosine phosphatase inhibition assay: IC_50_ = 5.2 μM. DPPH radical scavenging activity: IC_50_ = 93.94 μM.	[78,82,84,86,91,92,93,94,95,96,99,100,105,109,110,111,112,113,114,115,116]
Lantadene A acyl chloride * (**58**)	In vitro cytotoxic activity towards HL-60 cells: IC_50_ = 47.79 ± 0.24 μM; Hela cells: IC_50_ = 46.21 ± 0.17 μM; colon 502,713 cells: IC_50_ = 49.19 ± 0.17 μM; lung A549 cells: IC_50_ = 50.07 ± 0.14 μM.	[93]
Lantadene A methyl ester * (**81**)	In vitro cytotoxic activity towards HL-60 cells: IC_50_ = 34.04 ± 0.26 and 34 ± 1.4 μM; Hela cells: IC_50_ = 37.93 ± 0.09 and 37 ± 5 μM; colon 502,713 cells: IC_50_ = 37.22 ± 0.15 and 37 ± 8 μM; lung A549 cells: IC_50_ = 33.87 ± 0.09 and 33 ± 5 μM. In vivo antitumor activity: squamous cell carcinogenesis induced by 7,12-dimethylbenz[a]anthracene-12-*O*-tetradecanoylphorbol-13-*O*-acetate in Swiss albino mice (LACCA/female); 50 mg compound/kg body weight administered orally for 20 weeks: 87.5–100% mice survival and 13.6–19.6% overall papilloma incidence.	[112]
Lantadene A nitrile * (**57**)	In vitro cytotoxic activity towards HL-60 cells: IC_50_ = 70.43 ± 0.22 μM; Hela cells: IC_50_ = 74.0 ± 0.09 μM; colon 502,713 cells: IC_50_ = 78.68 ± 0.15 μM; lung A549 cells: IC_50_ = 82.80 ± 0.18 μM. In vivo antitumor activity: squamous cell carcinogenesis induced by 7,12-dimethylbenz[a]anthracene-12-*O*-tetradecanoylphorbol-13-*O*-acetate in Swiss albino mice (LACCA/female); 50 mg compound/kg body weight given orally for 20 weeks: 75% mice survival and 24.9% overall papilloma incidence.	[93]
**Lantadene B** (**61**)	Phytotoxic activity against *Eichhornia crassipes* (Mart.) Solms and *Microcystis aeruginosa* Kütz: ErC_50_ = 19.53 and 17.37 mM, respectively. Binding affinity to the antiapoptotic protein Bcl-xL: Ki > 100 μM. In vitro cytotoxic activity against KB cells: IC_50_ = 25.3 μM; HCT-116 cells: IC_50_ = 11.4 μM; MCF-7 cells: IC_50_ = 44 μM and ˃ 100 μM; L1210 cells: IC_50_ = 16.1 μM; A549 (lung carcinoma) cells: IC_50_ = 1.19 ± 0.28 μM. In vitro cytotoxic activity (MTT test) towards MCF-7 breast cancer cells: IC_50_ = 1.13 μM. In vitro anti-inflammatory activity (inhibition of TNF-α-induced NF-ΚB activation): IC_50_ = 1.56 ± 0.04 μM; COX-2 inhibition: IC_50_ ˃ 100 μM. In vitro nematocidal activity against *Meloidogyne incognita* larvae: 60% mortality at 0.25% concentration after 48 h; against *Leishmania mexicana* promastigotes, IC_50_ = 23.45 ± 2.15 μM. In vitro protein tyrosine phosphatase inhibition assay: IC_50_ = 5.5 μM. DPPH radical scavenging activity: IC_50_ = 76.45 μM.	[82,84,95,96,109,110,116,117,124]
**Lantadene C** (**72**)	Binding affinity to the antiapoptotic protein Bcl-xL: Ki > 100 μM. In vitro cytotoxic activity towards KB cells: IC_50_ = 15.8 μM; HCT-116 cells: IC_50_ = 41.8 μM; MCF7 cells: IC_50_ = 44.7 and ˃ 100 μM; L1210 cells: IC_50_ = 16.3 μM; HL-60 cells: IC_50_ ˃ 100 μM; Hela cells: IC_50_ ˃ 100 μM; colon 502,713 cells: IC_50_ ˃ 100 μM; lung A549 cells: IC_50_ ˃ 100 μM. DPPH radical scavenging activity: IC_50_ ˃ 100 μM.	[84,116]
**Lantadene D** (**51**)	In vivo antitumor activity: squamous cell carcinogenesis induced by 7,12-dimethylbenz[a]anthracene-12-*O*-tetradecanoylphorbol-13-*O*-acetate in Swiss albino mice (LACCA/female); 50 mg compound/kg body weight given orally for 20 weeks: approximately 85% mice survival and 30% overall papilloma incidence. In vitro protein tyrosine phosphatase inhibition assay: IC_50_ = 7.9 μM [84].	[84,97]
**Lantanilic acid** (**68**)	In vitro nematocidal activity against *Meloidogyne incognita* larvae: 98.66% mortality at 0.5% concentration after 48 h. In vitro antiparasitic activity against *Leishmania mexicana* promastigotes: IC_50_ = 9.50 ± 0.28 μM; *L. major* promastigotes: IC_50_ = 21.3 ± 0.02 μM; brine shrimp toxicity assay: LC_50_ = 49.20 μM. In vitro antibacterial and antifungal activity: diameter of inhibition zone at a concentration of 500 μg/mL against *S. aureus* = 1.7 mm and against *C. albicans* = 9.3 mm. In vitro protein tyrosine phosphatase inhibition assay: IC_50_ = 7.5 μM [84]. In vitro cytotoxic activity towards HL-60 human promyelocytic leukemia cells (JCRB0085): IC_50_ = 4.00 ± 0.67 μM. In vitro anti-inflammatory activity (inhibition of LPS-induced NO production in BV-2 cells): IC_50_ > 3 μM.	[78,80,81,84,98,99,118,122,123]
**Lantaninilic acid** (**30**)	In vitro antiparasitic activity against *Leishmania major* promastigotes: IC_50_ = 164 ± 0.8 μM. In vitro nematocidal activity against *Meloidogyne incognita* larvae: 60% mortality at a concentration of 0.125% after 48 h. In vitro cytotoxic activity towards HL-60 human promyelocytic leukemia cells (JCRB0085): IC_50_ = 68.4 ± 15.4 μM.	[78,82]
**Lantanolic acid** (**27**)	In vitro nematocidal activity against *Meloidogyne incognita* larvae: 100% mortality at a concentration of 1 mg/mL after 24 h. In vitro protein tyrosine phosphatase inhibition assay: IC_50_ = 13 μM.	[84,90]
**Lantanoside** (**140**)	In vitro nematocidal activity against *Meloidogyne incognita* larvae: 95% mortality at a concentration of 1% after 48 h. In vitro antibacterial activity against *Mycobacterium tuberculosis* strain H37Rv (ATCC 27294): 37% inhibition, MIC > 12.05 μM.	[16,134]
**Lantic acid** (**102**)	In vitro antimicrobial activity (bioautography assays): minimum growth inhibition values for *B. subtilis* (ATCC 6633), *M. luteus* (ATCC 9341), *S. aureus* (ATCC 6538P), and *P. mirabilis* (ATCC 14153) = 0.3 μg; *B. cereus* (ATCC 11778) = 0.1 μg; *S. faecalis* (ATCC 8043) and *P. aeruginosa* (ATCC 25619) = 1.1 nmol; *E. coli* (ATCC 25922) = 0.17 nmol. In vitro nematocidal activity against *Meloidogyne incognita* larvae: 100% mortality at a concentration of 1 mg/mL after 24 h.	[90]
**Lantoic acid** (**105**)	In vitro nematocidal activity against *Meloidogyne incognita* larvae: 100% mortality at a concentration of 1 mg/mL after 24 h. In vitro antiparasitic activity against *Leishmania major* promastigotes: IC_50_ = 97 ± 0.02 μM.	[81,90]
**Lantrieuphpene A** (**13**)	In vitro anti-inflammatory activity (inhibition of LPS-induced NO production in BV-2 cells): IC_50_ > 30 μM.	[80]
**Lantrieuphpene B** (**8**)	In vitro anti-inflammatory activity: inhibition of LPS-induced NO production in BV-2 cells, IC_50_ = 24 ± 0.30 μM; ROS and NO levels in LPS-stimulated zebrafish embryos significantly decreased in a concentration-dependent manner. Western blotting: iNOS protein expression decreased in a dose-dependent manner on pretreated cells.	[80]
**Lantrieuphpene C** (**9**)	In vitro anti-inflammatory activity: inhibition of LPS-induced NO production in BV-2 cells, IC_50_ = 27.98 ± 0.98 μM; ROS and NO levels in LPS-stimulated zebrafish embryos significantly decreased in a concentration-dependent manner. Western blotting: iNOS protein expression decreased in a dose-dependent manner on pretreated cells.	[80]
**Lantrieuphpene D** (**12)**	In vitro anti-inflammatory activity: inhibition of LPS-induced NO production in BV-2 cells, IC_50_ > 10 μM.	[80]
**Linaroside** (**139**)	In vitro nematocidal activity against *Meloidogyne incognita* larvae: 90% mortality at a concentration of 1% after 48 h. In vitro antibacterial activity against the *Mycobacterium tuberculosis* strain H37Rv (ATCC 27294): 30% inhibition, MIC = 13.12 μM. In vitro antioxidant activity (DPPH test): IC_50_ = 149.09 mM.	[16,134,162]
Methyl 22*β*-acetyloxy-oleanonate * (**45**)	In vitro cytotoxic activity towards HL-60 cells: IC_50_ = 72.75 ± 0.29 μM; Hela cells: IC_50_ = 70.6 ± 0.10 μM; colon 502,713 cells: IC_50_ = 67.48 ± 0.15 μM; lung A549 cells: IC_50_ = 71.77 ± 0.10 μM.	[94]
**Methyl 22*β*-angelyloxy-2-hydroxy-3-oxo-olean-1,12-diene-28-oate** (**78**)	In vitro cytotoxic activity towards HL-60 cells: IC_50_ = 26 ± 6 μM; HeLa cells: IC_50_ = 31 ± 5 μM; colon 502,713 cells: IC_50_ = 32 ± 1 μM; lung A549 cells: IC_50_ = 28 ± 4 μM. In vivo antitumor activity: squamous cell carcinogenesis induced by 7,12-dimethylbenz[a]anthracene-12-*O*-tetradecanoylphorbol-13-*O*-acetate in Swiss albino mice (LACCA/female); 50 mg compound/kg body weight administered orally for 20 weeks: approximately 100% mice survival and 17.2% overall papilloma incidence.	[124]
Methyl 22*β*-benzoyloxy-oleanonate * (**86**)	In vitro cytotoxic activity towards HL-60 cells: IC_50_ = 81.52 ± 0.08 μM; Hela cells: IC_50_ = 86.10 ± 0.08 μM; colon 502,713 and lung A-549 cells: IC_50_ > 100 μM.	[94]
Methyl 22*β*-butanoyloxy-oleanonate * (**73**)	In vitro cytotoxic activity towards HL-60 cells: IC_50_ = 34.79 ± 0.14 μM; Hela cells: IC_50_ = 36.23 ± 0.38 μM; colon 502,713 cells: IC_50_ = 38.03 ± 0.09 μM; lung A549 cells: IC_50_ = 40.37 ± 0.09 μM. In vivo antitumor activity: squamous cell carcinogenesis induced by 7,12-dimethylbenz[a]anthracene-12-*O*-tetradecanoylphorbol-13-*O*-acetate in Swiss albino mice (LACCA/female); 50 mg compound/kg body weight administered orally for 20 weeks: 80% mice survival and 12.4% overall papilloma incidence.	[94]
Methyl 22*β*-hydroxy-oleanonate * (**35**)	In vitro cytotoxicity towards HL-60 cells: IC_50_ ˃ 100 μM; Hela cells: IC_50_ > 100 μM; colon 502,713 cells: IC_50_ ˃ 100 μM; lung A549 cells: IC_50_ ˃ 100 μM.	[91,94]
Methyl 22*β*-isobutyryloxy-oleanonate * (**74**)	In vitro cytotoxicity towards HL-60 cells: IC_50_ = 71.19 ± 0.09 μM; Hela cells: IC_50_ = 74.08 ± 0.38 μM; colon 502,713 cells: IC_50_ = 68.67 ± 0.09 μM; lung A549 cells: IC_50_ = 76.06 ± 0.14 μM.	[94]
Methyl 22*β*-propanoyloxy-oleanonate * (**52**)	In vitro cytotoxicity towards HL-60 cells: IC_50_ = 44.75 ± 0.39 μM; Hela cells: IC_50_ = 48.81 ± 0.15 μM; colon 502,713 cells: IC_50_ = 41.42 ± 0.15 μM; lung A549 cells: IC_50_ = 52.52 ± 0.39 μM. In vivo antitumor activity: squamous cell carcinogenesis induced by 7,12-dimethylbenz[a] anthracene-12-*O*-tetradecanoylphorbol-13-*O*-acetate in Swiss albino mice (LACCA/female); 50 mg compound/kg body weight administered orally for 20 weeks: 80% mice survival and 17.9% overall papilloma incidence.	[94]
**Oleanolic acid** (**31**)	In vitro larvicidal activity: 30% lethality in the brine shrimp lethality test against *Spodoptera littoralis* Biosduval after 48 h at a concentration of 10.95 mM. Inactive in the fecundity inhibition assay against *Clavigralla tomentosicollis* Stal. and *Aphis craccivora* Koch. In vitro nematocidal activity against *Meloidogyne incognita* larvae: 70.33% mortality at a concentration of 0.5% after 48 h. In vitro antifilarial activity against *Brugia malayi*: LC_100_ = 136.85 μM. In vivo antifilarial activity against *Brugia malayi* in rodel model *Mastomys coucha* (100 or 200 mg compound/kg body weight administered intraperitoneally for 5 days): macrofilaricidal efficacy = 9.09% and 18.18%, respectively; percent female sterility = 49.22 ± 10.57 and 56.50 ± 9.50, respectively. In vitro cytotoxic activity towards HCT-15 cells: IC_50_ = 52 μM; SW-620 cells: IC_50_ = 25 μM; A549 cells: IC_50_ = 52 μM; IGROV-1 cells: IC_50_ = 8 μM; IMR-32 cells: IC_50_ = 61 μM. In vitro antiparasitic activity against *Leishmania major* promastigotes: IC_50_ = 53 ± 0.02 μM. In vitro protein tyrosine phosphatase inhibition assay: IC_50_ = 2 μM. In vitro anti-inflammatory activity (inhibition of LPS-induced NO production in BV-2 cells): IC_50_ > 60 μM.	[75,78,80,84,86,87,118]
**Oleanonic acid** (**22**)	In vitro larvicidal activity: 20% lethality in the brine shrimp lethality test against *Spodoptera littoralis* Biosduval: after 48 h at a concentration of 10.996 mM. Inactive in the fecundity inhibition assay towards *Clavigralla tomentosicollis* Stal. and *Aphis craccivora* Koch. In vitro antifilarial activity against *Brugia malayi*: LC_100_ = 68.73 μg/mL. In vivo antifilarial activity against *Brugia malayi* in rodel model *Mastomys coucha* (100 or 200 mg compound/kg body weight administered intraperitoneally for 5 days), macrofilaricidal efficacy: inactive; % female sterility: 56.56 ± 9.49 and 29.71 ± 6.52, respectively. In vitro cytotoxic activity towards EAC cells: IC_50_ = 7.1 ± 1.3 μM; A375 cells: IC_50_ = 10.9 ± 1.5 μM; Hep2 cells: IC_50_ = 59.3 ± 1.1 μM; U937 cells: IC_50_ = 16.5 ± 1.3 μM; HL-60 human promyelocytic leukemia cells (JCRB0085): IC_50_ = 9.79 ± 2.13 μM; PMBC cells: IC_50_ > 100 μM. In vitro nematocidal activity against *Meloidogyne incognita* larvae: 80% mortality at a concentration of 0.5% after 48 h. In vitro protein tyrosine phosphatase inhibition essay: IC_50_ = 6.9 μM [84].	[26,75,82,84,86,88,99]
**11-Oxo-*β*-boswellic acid** (**103**)	In vitro antifungal activity against *Fusarium subglutinans* (PPRI 6740) and *F. semitectum* (PPRI 6739): MIC = 1.338 mM; *F. proliferatum* (PPRI 18679): MIC = 2.762 mM; *F. solani* (PPRI 19147) and *F. graminearum* (PPRI 10728): MIC = 5.311 mM. In vitro cytotoxicity towards Raw 264.7 cells: IC_50_ ˃ 100 μM.	[120]
**Pomolic acid** (**107**)	In vitro nematocidal activity against *Meloidogyne incognita* larvae: 100% mortality at a concentration of 1 mg/mL after 24 h. In vitro protein tyrosine phosphatase inhibition assay: IC_50_ = 10.6 μM.	[84,90]
**Pomonic acid** (**104**)	In vitro protein tyrosine phosphatase inhibition assay: IC_50_ = 10.5 μM. In vitro anti-inflammatory activity (inhibition of LPS-induced NO production in BV-2 cells): IC_50_ > 30 μM.	[80,84]
**22*β*-Propanoyl-** oxy-oleanonic acid * (**46**)	In vitro cytotoxic activity towards HL-60 cells: IC_50_ = 50.12 ± 0.32 μM; Hela cells: IC_50_ = 54.29 ± 0.09 μM; colon 502,713 cells: IC_50_ = 48.22 ± 0.09 μM; lung A549 cells: IC_50_ = 56.19 ± 0.26 μM. In vivo antitumor activity: squamous cell carcinogenesis induced by 7,12-dimethylbenz[a] anthracene/12-*O*-tetradecanoylphorbol-13-*O*-acetate in Swiss albino mice (LACCA/female); 50 mg compound/kg body weight administered orally for 20 weeks: 80% mice survival and 19.6% overall papilloma incidence.	[94]
**Reduced lantadene A** (22*β*-angelyloxy-3*β*-hydroxy-olean-12-en-28-oic acid) (**75**)	Evaluation of toxicity to sheep: 80 mg compound/kg body weight administered orally in gelatin capsules: nontoxic; 80 mg compound/kg body weight, dissolved in DMSO, intraruminal administration: toxic. Evaluation of toxicity to Wistar female rats: 15 mg compound/kg body weight administered orally in olive oil: toxic. In vitro antitumor activity: Epstein–Barr virus early antigen activation assay induced by 12-*O*-tetradecanoylphorbol-13-*O*-acetate (TPA) in Raji cells: 30.6% inhibition at a concentration of 100 mol compound/1 mol TPA. In vitro cytotoxicity was tested towards multiple cancer cells. In vitro protein tyrosine phosphatase inhibition assay: IC_50_ = 7.2 μM.	[84,91,95]
**Reduced lantadene B** (3*β*-hydroxy-22*β*-senecioyloxy-olean-12-en-28-oic acid) (**76**)	In vitro cytotoxicity was tested towards multiple cancer cells. In vitro protein tyrosine phosphatase inhibition assay: IC_50_ = 5.1 μM.	[95]
**Reduced lantadene C** 3*β*-hydroxy-22*β*-[2-methylbutanoyloxy]-olean-12-en-28-oic acid (**77**)	In vitro protein tyrosine phosphatase 1B inhibition assay: IC_50_ = 7.3 μM.	[84]
***β*-Sitosterol** (**3**)	In vitro antiparasitic activity against *Brugia malayi*: LC_100_ > 1.2 mM. Antibacterial activity (disk diffusion method): diameter of inhibition zone = 14 mm for *Escherichia coli*, 19 mm for *Staphylococcus aureus*, 17 mm for *Salmonella typhimurium*, 24 mm for *Pseudomonas aeruginosa*. The cytotoxic potential was tested in vitro by an MTT assay against T47D (breast cancer cells) and HeLa (cervical cancer cells): IC_50_ = 24.06 and 24.86 µM, respectively.	[73,74,170]
***β*-Sitosterol 3-*O*-β-D-glucopyrano-side** (**4**)	In vitro antiparasitic activity against *Brugia malayi*: LC_100_ > 0.86 mM.	[71]
**Stearic acid** (**158**)	In vitro antiparasitic activity against *Brugia malayi:* LC_100_ > 1.7 mM.	[74,77]
**Trilinolein** (**168**)	In vitro antibacterial activity (disk diffusion method): diameter of inhibition zone = 20 mm for *Escherichia coli*, 19 mm for *Staphylococcus aureus*, 18 mm for *Salmonella typhimurium*, 21 mm for *Pseudomonas aeruginosa*.	[73]
**Urs-12-en-3*β*-ol-28-oic acid 3-*O*-β-D-glucopyrano- syl-4′-octadecano- ate** (***127***)	In vivo antidiabetic activity: Wistar albino rats (150–200 g) received 0.3 mg/kg body weight orally for 21 days. Blood glucose levels: 8th day = 183.56 ± 3.61 mg/dL, 14th day = 143.43 ± 2.79 mg/dL, 21st day = 118.67 ± 2.40 mg/dL. In vivo anxiolytic activity: dose-dependent effect.	[125]
**Ursolic acid** (**106**)	In vitro nematocidal activity against *Meloidogyne incognita* larvae: 100% mortality at a concentration of 1 mg/mL after 48 h. In vitro antiparasitic activity against *Leishmania major* promastigotes: IC_50_ = 12.4 ± 0.03 μM.	[78,90]

^a^ Compounds are ordered in alphabetic order. Cytotoxicity values (IC_50_) are expressed in μM for homogeneity. * Semisynthetic derivative. ErC_50_ = concentration of test substance which caused 50% reduction in growth rate relative to the control for a 72 h exposure.

A few compounds isolated from *L. camara* were also submitted to molecular docking studies (Table 10) towards the active site of RNA-dependent RNA polymerase (RdRp) of SARS-CoV-2. The highest binding energy (around 6 kcal/mol) was determined for camarolic acid (**69**) and lantoic acid (**105**). These values, when compared to remdesivir (−5.75 kcal/mol), indicated that compounds **69** and **105** can serve as promising anti-COVID-19 candidates. Moreover, lantrieuphpene B (**8**) and C (**9**) exhibited a high binding affinity (a binding energy of around 9 kcal/mol) to the TYR-341, TYR-367, and ASP-376 residues of inducible Nitric Oxide Synthase (iNOS). In addition, a recent in silico study has evaluated 20 selected constituents of *L. camara* as potent inhibitors of the human enzymes acetylcholinesterase (hAchE), carbonic anhydrase II (hCA-II), and carboxylesterase 1 (hCES-1), which are pharmacological targets for the treatment of neurodegenerative diseases, glaucoma, obesity, and type 2 diabetes [171]. All of the twenty ligands docked effectively with the CA-II enzyme. Only ursonic acid (**100**) was ineffective in both docking and binding with AchE and CES-1, while lantic acid (**102**) exhibited the least atomic binding energy with all three enzymes. The glucosyl flavone camaroside [17] exhibited the maximum binding energy (−9.34 kcal/mol) with hAchE, while the phenylethanoid glycoside isonuomioside A [17] demonstrated the highest binding energy (−9.72 kcal/mol) with hCA-II, and the flavone pectolinarin (**146**) showed the highest binding energy (−9.21 kcal/mol) with hCES-1 [172].

**Table 10:** In silico studies of compounds isolated from *Lantana camara*.

Compound (Nº)	Docking Value	Reference
**Camaranoic acid** (**99**)	Molecular docking into the active site of SARS-CoV-2 RNA-dependent RNA polymerase (RdRp): binding free energy = 1.272 kcal/mol.	[81]
**Camaric acid** (**62**)	Molecular docking into the active site of SARS-CoV-2 RdRp: binding free energy = −3.198 kcal/mol.	[81]
**Camarolic acid** (**69**)	Molecular docking into the active site of SARS-CoV-2 RdRp: binding free energy = −6.73 kcal/mol.	[81]
**Icterogenin** (**67**)	Molecular docking into the active site of SARS-CoV-2 RdRp: binding energy = −2.311 kcal/mol.	[81]
**Lantabetulic acid** (**17**)	Molecular docking into the active site of SARS-CoV-2 RdRp: binding free energy = −2.958 kcal/mol.	[81]
**Lantacin** (**119**)	Molecular docking into the active site of SARS-CoV-2 RdRp: binding free energy = −2.919 kcal/mol.	[81]
**Lantaiursolic acid** (**120**)	Molecular docking into the active site of SARS-CoV-2 RdRp: binding free energy = −3.867 kcal/mol.	[81]
**Lantanilic acid** (**68**)	Molecular docking into the active site of SARS-CoV-2 RdRp: binding free energy = −3.633 kcal/mol.	[81]
**Lantoic acid** (**105**)	Molecular docking into the active site of SARS-CoV-2 RdRp: binding free energy = −6.07 kcal/mol.	[81]
**Lantrieuphpene B** (**8**)	Binding free energy = −9.8 Kcal/mol with TYR-341, TYR-367, and ASP-376 residues of iNOS (inducible Nitric Oxide Synthase).	[80]
**Lantrieuphpene C** (**9**)	Molecular binding free energy = −9.3 Kcal/mol with TYR-341, TYR-367, and ASP-376 residues of iNOS.	[80]
***β*-Sitosterol** (**3**)	Molecular binding free energies towards Bcl-2 and HPV16 E7 protein receptors: −8.11 and −7.276 kcal/mol, respectively	[170]

Finally, in unusual applications, leaf, fruit, flower, root, and seed extracts of *Lantana camara* have been used to prepare several metal (Ag, Au, Fe, Cu, Zn, Pd, and Pt) [173] and metal oxide (ZnO, SrO, CuO, NiO, and Y_2_O_3_) nanoparticles with potential photocatalytic, electrochemical, anticancer, antiarthritic, and antibacterial properties, and other medical applications. The biomass and leaves of *L. camara* have also been used as a sustainable alternative for the removal of antibiotics and metals, such as Pb (II), Zn (II), and Mn (II) from contaminated rivers and waste waters [171,174,175,176].

## 4. Conclusions

This review, reporting on the recently published information on the phytochemistry and bioactivities of *L. camara*, clearly demonstrates that this species continues to be one of the most investigated plants due to the various traditional uses, the rich phytochemical contents of the extracts, and the wide variety of biological activities exhibited by total extracts, several isolated compounds, and the many semisynthetic derivatives.

*Perspectives*. In our opinion, among the various biological effects exhibited by specialized metabolites from *L. camara* (Table 8 and Table 9), the nematocidal and antiparasitic properties of several compounds and the antimalarial effects of leaf extracts against the chloroquine-sensitive strains 3D7 and D10, and the chloroquine-resistant strain W2, of *Plasmodium falciparum* deserve further investigations with in vivo and in the field tests. Given the various structural features of active compounds, there may also be an opportunity to conduct QSAR studies and to clarify the mechanism(s) of action, and to identify the molecular target(s) and the biological processes involved in the nematocidal and antiparasitic properties. Moreover, the interesting antidiabetic and anti-COVID-19 properties in vitro of leaf extracts and a few isolated compounds must be confirmed by additional in silico and in vivo studies. Computational strategies involving artificial intelligence and machine learning algorithms are expected to help in the full exploration of the biological space of natural molecules from *L. camara*, and to identify the unexplored human receptors and enzymes to which they can bind. Semisynthesis is an important technique to harness nature’s diversity for novel drugs. In this regard, semisynthetic efforts to prepare analogs of natural products isolated from *L. camara* to enhance their biological properties are limited and, therefore, they must be intensified.

Finally, preclinical and clinical research studies, which are missing so far, are necessary to evaluate the efficacy and safety of the products with the most promising medicinal properties.

## Data Availability

All data are available on database reported.
